# Interleukin-6 *Trans*-Signaling Pathway Promotes Immunosuppressive Myeloid-Derived Suppressor Cells *via* Suppression of Suppressor of Cytokine Signaling 3 in Breast Cancer

**DOI:** 10.3389/fimmu.2017.01840

**Published:** 2017-12-15

**Authors:** Mengmeng Jiang, Jieying Chen, Wenwen Zhang, Rui Zhang, Yingnan Ye, Pengpeng Liu, Wenwen Yu, Feng Wei, Xiubao Ren, Jinpu Yu

**Affiliations:** ^1^Cancer Molecular Diagnostics Core, Tianjin Medical University Cancer Institute and Hospital, National Clinical Research Center for Cancer, Key Laboratory of Cancer Prevention and Therapy, Tianjin’s Clinical Research Center for Cancer, Tianjin, China; ^2^Department of Immunology, Tianjin Medical University Cancer Institute and Hospital, National Clinical Research Center for Cancer, Key Laboratory of Cancer Immunology and Biotherapy, Tianjin’s Clinical Research Center for Cancer, Tianjin, China; ^3^Department of Urology, Tianjin Medical University General Hospital, Tianjin, China

**Keywords:** breast cancer, interleukin-6, myeloid-derived suppressor cells, suppressor of cytokine signaling 3, the JAK/STAT signaling pathway, *trans*-signaling pathway

## Abstract

Interleukin-6 (IL-6) has been reported to stimulate myeloid-derived suppressor cells (MDSCs) in multiple cancers, but the molecular events involved in this process are not completely understood. We previously found that cancer-derived IL-6 induces T cell suppression of MDSCs *in vitro via* the activation of STAT3/IDO signaling pathway. In this study, we aimed to elucidate the underlying mechanisms. We found that in primary breast cancer tissues, cancer-derived IL-6 was positively correlated with infiltration of MDSCs *in situ*, which was accompanied by more aggressive tumor phenotypes and worse clinical outcomes*. In vitro* IL-6 stimulated the amplification of MDSCs and promoted their T cell suppression ability, which were fully inhibited by an IL-6-specific blocking antibody. Our results demonstrate that IL-6-dependent suppressor of cytokine signaling 3 (SOCS3) suppression in MDSCs induced phosphorylation of the JAK1, JAK2, TYK2, STAT1, and STAT3 proteins, which was correlated with T cell suppression of MDSCs *in vitro*. Therefore, dysfunction in the SOCS feedback loop promoted long-term activation of the JAK/STAT signaling pathway and predominantly contributed to IL-6-mediated effects on MDSCs. Furthermore, IL-6-induced inhibition of SOCS3 and activation of the JAK/STAT pathway was correlated with an elevated expression of IL-6 receptor α (CD126), in which the soluble CD126-mediated IL-6 *trans*-signaling pathway significantly regulated IL-6-mediated effects on MDSCs. Finally, IL-6-induced SOCS3 dysfunction and sustained activation of the JAK/STAT signaling pathway promoted the amplification and immunosuppressive function of breast cancer MDSCs *in vitro* and *in vivo*, and thus blocking the IL-6 signaling pathway is a promising therapeutic strategy for eliminating and inhibiting MDSCs to improve prognosis.

## Introduction

Increasing evidence has highlighted the importance of crosstalk between cancer cells and the surrounding microenvironment in the initiation and progression of various cancers (1–3). The tumor microenvironment is composed of multiple immunosuppressive cells, among which myeloid-derived suppressor cells (MDSCs) play a vital role in promoting tumor invasion and metastasis (3).

Myeloid-derived suppressor cells are a heterogeneous population of immature myeloid cells with suppressive effects on both innate and adaptive immunity; therefore, they are regarded as a major obstacle in antitumor immunotherapy (4). Different MDSCs subsets display varied phenotypes in mice or in humans. For example, monocytic MDSCs (M-MDSCs) express CD11b^+^Ly6G^−^Ly6C^hi^ in mice and CD11b^+^HLA-DR^−/lo^CD14^+^CD15^−^ in human; while polymorphonuclear MDSCs (PMN-MDSCs) express CD11b^+^Ly6G^+^Ly6C^lo^ in mice and CD11b^+^HLA-DR^−/lo^CD14^−^CD15^+^ in human. Additionally, early-stage MDSCs (eMDSCs), which comprised more immature progenitors than M-MDSCs and PMN-MDSCs, are defined as specific MDSCs subset expressing Lin^−^HLA-DR^−^CD33^+^ in human tumors (5). In our previous study, we identified a subset of poorly differentiated eMDSCs in breast cancer that expressed an immature phenotype of Lin^−^HLA-DR^−^CD45^+^CD33^+^CD13^+^CD14^−^CD15^−^ and displayed potent suppression of T cells *in vitro* and *in vivo* (6). We found that cancer-derived interleukin-6 (IL-6) induces the immunosuppressive ability of MDSCs by activating the STAT3/IDO signaling pathway, but the detailed molecular events are unclear (7).

Interleukin-6 is known as a key regulator of immunosuppression in advanced cancer and is responsible for the development of pro-inflammatory and metastatic tumor microenvironments (8). Numerous studies have reported significant correlations between IL-6 and circulating MDSCs in both human and mouse models (9–14). IL-6 increased circulating CD11b^+^CD14^+^HLA^−^DR^−^ cells in squamous carcinoma of the esophagus (9) and prostate cancer (13). Though previous studies reported that IL-6 restored MDSC accumulation in a mouse model of mammary carcinoma (14), a few studies have focused on the relationship between IL-6 and MDSCs in human breast cancer. Our previous study demonstrated that in breast cancers, IL-6 stimulates STAT3-dependent, nuclear factor-κB-mediated indoleamine 2,3-dioxygenase (IDO) upregulation in MDSCs (7); this triggers immunosuppressive effects of MDSCs *in vitro* and *vivo* (6). Although abnormal accumulation of MDSCs *via* the IL-6/STAT3 pathway was reported in multiple cancers (9, 13, 15), the major regulatory mechanisms remain unclear.

It is well-established that the interaction between IL-6 and IL-6R initiates the activation of the JAK/STAT signaling pathway, which transduces the IL-6 signal in both normal and malignant cells. In contrast to the rapid and reversible activation of STAT proteins in normal cells, phosphorylation of STAT proteins is sustained for a long time in malignant cells (16, 17). The dysfunctional negative feedback loop in the JAK/STAT signaling pathway induces constitutive activation of STAT proteins, oncogenic transformation, tumor invasion, and metastasis (18).

Suppressor of cytokine signaling (SOCS) proteins, particularly SOCS3, are major negative feedback regulators of the JAK/STAT signaling pathway (19). Under physiological conditions, IL-6 stimulates the expression of SOCS3 and inhibits phosphorylation of STAT proteins (20). This attenuates IL-6-induced activation of the JAK/STAT signaling pathway and inhibits expression of downstream functional genes (17, 21). It has been reported that constitutive defects in the expression of SOCS3 protein is frequent in malignant cells and is associated with dysregulation of cell growth, migration, and apoptosis (19).

However, only short-term and reversible suppression of SOCS was detected in certain types of immune cells in cancer, such as tumor-infiltrated T cells, dendritic cells (DCs), and macrophages (22, 23). It has been demonstrated that knockdown of SOCS3 in macrophages is beneficial for inhibiting tumor metastases in mice (24). However, it has also been reported that SOCS3 deficiency in myeloid cells promotes tumor development by inducing MDSCs in the tumor microenvironment (25). Therefore, it is urgent to elucidate the biological significance of SOCS3 deficiency in MDSC development and tumor progression, which may provide insight into potential therapeutic targets for breast cancer.

In this study, we evaluated the expression of SOCS proteins and their effects on IL-6-induced activation of the JAK/STAT signaling pathway in breast cancer MDSCs. We found that more MDSCs were recruited in IL-6 high-expressing breast cancer tissues, in which SOCS3 inhibition was detected. IL-6 promoted the amplification of MDSCs and enhanced their suppressive effects on T cells immunity *in vitro*. IL-6 stimulated SOCS3 suppression and thus induced long-term activation of the JAK/STAT signaling pathway in breast cancer MDSCs. IL-6-induced SOCS3 dysfunction and sustained activation of the JAK/STAT signaling pathway predominantly contributed to IL-6-mediated effects on MDSCs. Furthermore, we observed that IL-6-induced suppression of SOCS3 and activation of the JAK/STAT pathway were correlated with an elevated expression of IL-6 receptor α (IL-6-Rα, CD126), which was regulated by the soluble CD126-mediated IL-6 *trans*-signaling pathway. Summarily, we found that IL-6-induced SOCS3 dysfunction and sustained activation of the JAK/STAT signaling pathway promoted the amplification and immunosuppressive function of breast cancer MDSCs *in vitro* and *vivo*. Thus, blocking IL-6 signaling pathway is a promising therapeutic strategy for eliminating and inhibiting MDSCs to improve prognosis.

## Materials and Methods

### Clinical Samples and Healthy Donors

In this study, we collected 253 primary breast cancer tissue samples from two cohorts for immunohistochemistry (IHC) analysis. Cohort 1 included 113 primary breast cancer patients who received surgical resection at the Department of Breast Oncology of Tianjin Medical University Cancer Institute and Hospital from October 2012 to October 2014. Cohort 2 included 140 breast cancer cases whose tumor tissues were assembled on tissue arrays after surgical removal between January 2001 and August 2004 from Shanghai Outdo Biotech Co., Ltd. (Shanghai, China). All patients were women with a median age of 52 years (29–79 years) in cohort 1 and 51 years (29–83 years) in cohort 2. Among the patients, infiltrated mammary-ductal carcinoma accounted for 78.8% (89/113) in cohort 1 and 93.6% (131/140) in cohort 2. Cohort 1 included 9, 72, and 26 patients with histological grade I, II, and III cancer, respectively. Cohort 2 included 19, 111, and 7 patients with histological grade I, II, and III cancer, respectively. Cohort 1 included 18, 65, and 29 patients with clinical stage I, II, and III cancer, respectively. Cohort 2 included 11, 80, and 47 patients with clinical stage I, II, and III cancer, respectively (Table 1). Three patients in cohort 2 were excluded from the study because of non-cancer related death.

**Table 1:** Baseline of all patients.

Baseline	Cohort 1	Cohort 2
Total	113	140
**Age**		
≤52 years	57	77
>52 years	55	63
**Pathology**		
iDC	89	131
Non-iDC	24	9
**Histology grade**		
0–I	9	19
II	72	111
III	26	7
**Lymph nodes**		
Negative	81	84
Positive	28	52
**Tumor size**		
≤3 cm	79	81
>3 cm	30	59
**Stage**		
0–I	18	11
II	65	80
III–IV	29	47
**ER**		
Negative	37	51
Positive	76	81
**PR**		
Negative	44	61
Positive	69	71
**HER2**		
Negative	62	95
Positive	29	37

In addition, 20 fresh primary breast cancer tissue samples and the corresponding adjacent tissues were obtained for isolation of primary MDSCs *in situ*. Peripheral blood (PB) samples (40) from healthy donors were collected to enrich CD3^+^ T cells to study the immunosuppressive ability of MDSCs *in vitro*. Additionally, CD33^+^ and CD14^+^ cells isolated from healthy donors’ PB samples were employed as myeloid progenitor and differentiated monocyte controls, respectively. This study was approved by the Medical Ethics Committee of Tianjin Medical University. All experiments were performed in accordance with the principles of the Declaration of Helsinki. Written consent was obtained from all patients and healthy donors.

### Isolation of Primary MDSCs and CD33^+^ Progenitors

Twenty primary breast cancer tissues and their adjacent tissues were collected during surgery and cut into small pieces before being ground and filtered using a filter mesh to prepare a single-cell suspension. CD33^+^ cells were isolated using human CD33 MicroBeads (130-045-501; Miltenyi Biotec, Bergisch Gladbach, Germany) as previously reported (6). The negative-selected cells were defined as breast cancer cells and analyzed for the expression of IL-6. Thirty cases of PB samples were collected from healthy donors for PBMC isolation. CD33^+^ and CD14^+^ cells were enriched using human CD33 (130-045-501; Miltenyi Biotec) and human CD14 MicroBeads (130-050-201; Miltenyi Biotec), respectively, according to the manufacturer’s instructions. Trypan blue staining was performed to ensure the full viability of each cell fraction.

### IHC Assay

Fresh tissues were immediately fixed with formalin after surgical removal; tissues were made into paraffin embedded blocks and then sliced into 4-µm serial sections. The samples were heated for 1 h at 70°C, deparaffinized in xylene, and rehydrated using graded alcohol. Antigens were retrieved in citrate buffer (pH 6.0) for 2 min. Endogenous peroxidase activity was quenched in 3% hydrogen peroxide for 20 min. We previously examined the expression a series of pan-myeloid and differentiated markers of myeloid linage and confirmed high expression of CD33 and CD13, low expression of HLA-DR and CD14, and negative expression of CD15 on the surface of breast cancer MDSCs (6). We also detected the expression of 3 pan-myeloid markers, including CD33 (26), CD13 (27), and CD11b (28) and found that non-specific staining of CD13 and CD11b on cancer cells, endothelial cells, and fibroblasts interfered with the specific staining on MDSCs (Figures S1B–C in Supplementary Material), indicating the feasibility of using CD33 to detect breast cancer MDSCs in an IHC assay. Therefore, all samples were incubated with mouse anti-human IL-6 (PeproTech, Rocky Hill, NJ, USA) and CD33 (Abcam, Cambridge, UK) monoclonal antibody (McAb) at a concentration of 1 µg/mL overnight at 4°C. A secondary antibody conjugated with streptavidin-horseradish peroxidase (Santa Cruz, Biotech, Dallas, TX, USA) was then added, and the mixture was incubated for 30 min before adding diaminobenzidine staining buffer (Maixin Biotechnology, Fuzhou, China). All images were captured using an Olympus BX51 microscope (Olympus, Tokyo, Japan). Five representative high-power fields (400× magnification) from each tissue section were selected for histology evaluation as previously described (6).

### Flow Cytometry Analysis

Primary MDSCs were isolated from primary breast tumor tissues. To assess the proportions of CD45^+^CD13^+^CD33^+^CD14^−^CD15^−^ MDSCs in cancerous and corresponding adjacent normal tissues, flow cytometry analysis was performed using a BD FACS Canto™ II flow cytometer (BD Biosciences, San Jose, CA, USA). The PerCp-conjugated anti-human CD45, phycoerythrin-conjugated anti-human CD13 and CD33, and fluorescein isothiocyanate-conjugated anti-human CD14 and CD15 (BD Biosciences) antibodies were used to label the MDSCs. An isotype-matched IgG1 antibody (BD Biosciences) was used as a negative control. After incubation, cells were washed and resuspended in buffer, and the expression of cell surface markers was detected using the flow cytometer. The leukocyte population was gated using PerCp-labeled anti-human CD45, and breast cancer MDSCs were defined as CD33^+^CD13^+^CD14^−^CD15^−^ in the CD45^+^ population. Furthermore, to detect the production of interferon (IFN)-γ and IL-10 in T cells co-cultured with or without MDSCs, we conducted an intracellular staining assay using flow cytometry. After co-culture, T cells were distinguished by allophycocyanin-labeled anti-CD3 McAb and the percentages of IFN-γ positive and IL-10 positive T cells were determined using PE/Cy7-labeled anti-IFN-γ and PE-labeled anti-IL-10 McAbs, respectively.

### Cell Line and Cell Culture

The human breast cancer cell line MDA-MB-231 was obtained from the Chinese Academy of Medical Sciences (Resource number: 3111C0001CCC000014). The cell line was cultured in complete RPMI 1640 medium (Gibco BRL, Grand Island, NY, USA) containing 10% fetal bovine serum in a 5% CO_2_ incubator at 37°C. CD33^+^ progenitors isolated from PBMCs of healthy donors were co-cultured with breast tumor cells to induce MDSCs with or without IL-6 antibody. CD33^+^ and CD14^+^ control cells were cultured in complete RPMI 1640 medium. After co-culture with MDSCs for 3 days, the proliferation, apoptosis, and cytokine secretion of T cells were studied to evaluate the immunosuppressive ability of MDSCs pretreated with or without IL-6 antibody.

### Induction of MDSCs *In Vitro*

CD33^+^ myeloid progenitors (2 × 10^6^/mL) isolated from healthy PBMCs were added to multi-well plates and co-cultured with MDA-MB-231 breast cancer cells to induce MDSCs with or without IL-6 antibody (EMD Millipore, Billerica, MA, USA) at a concentration of 50 µg/mL. CD33^+^ progenitors were cultured in RPMI 1640 medium supplemented with 10% fetal bovine serum as negative controls. After 48 h of culture, MDSCs were harvested for further analysis, and the supernatants were collected to detect soluble CD126 using the Human IL-6R ELISA Kit (GenWay Biotech, Inc., San Diego, CA, USA). The phenotype of harvested cells was examined by flow cytometry as previously described; the proportion of CD45^+^CD13^+^CD33^+^CD14^−^CD15^−^ MDSCs was examined (6).

### Cell Counting Kit 8 (CCK8) Assay

Cell Counting Kit 8 assay was used to detect the proliferation of T cells co-cultured with or without MDSCs. CD3^+^ T cells were isolated from PBMCs of 10 healthy donors using the Human Pan T cell Isolation Kit II (130-091-156; Miltenyi Biotec). Both MDSCs and T cells with viability >95% were used for functional assays. Purified T cells (2 × 10^5^) were plated in a 96-well plate and co-cultured with CD33^+^ cells or MDSCs in the presence or absence of IL-6 antibody at ratio of 1:3 in triplicate. Cells were cultured in complete medium supplemented with 1,000 IU/mL recombinant human IL-2 (PeproTech) or anti-CD3/CD28 Abs (at bead/cell ratio of 1:1, Gibco) at 37°C in a 5% CO_2_ incubator for 3 days. Next, 10 µL CCK8 (Dojindo Molecular Technologies, Inc., Rockville, MD, USA) was added to each well and incorporated into living cells during cell proliferation. Blank wells without cells were used as negative controls. T cells stimulated with IL-2 or anti-CD3/CD28 Abs were used as the T cell control. After 4 h of incubation, the optical density at 450 nm was measured using an enzyme immunoassay analyzer (Thermo Fisher Scientific, Waltham, MA, USA). Cell proliferation was evaluated using stimulation index (SI), which was calculated using the following formula: SI = [(experimental counts)/(responder control counts + stimulator control counts)].

### Annexin V Assay

Purified T cells (5 × 10^5^) were plated in a 24-well plate and co-cultured with CD33^+^ cells or MDSCs in the presence or absence of IL-6 antibody at ratio of 1:3 in triplicate. Cells were cultured in complete medium supplemented with 1,000 IU/mL recombinant human IL-2 at 37°C in a 5% CO_2_ incubator for 3 days. The Annexin V assay was used to detect the apoptosis of T cells. We initially gated lymphocytes according to SSC and FSC features and then gated T cells using allophycocyanin-labeled anti-CD3 McAb (BioLegend, San Diego, CA, USA). The cells were stained with FITC-Annexin V and propidium iodide provided in an Apoptosis Detection Kit (BD Biosciences) as previously described (6). The positive expression of Annexin V and negative expression of PI represent apoptotic T cells.

### Enzyme-Linked Immunosorbent Assay (ELISA)

Purified T cells (5 × 10^5^) were plated in a 24-well plate and co-cultured with CD33^+^ cells or MDSCs in the presence or absence of IL-6 antibody at ratio of 1:3 in triplicate. Cells were cultured in complete medium supplemented with 1,000 IU/mL recombinant human IL-2 or anti-CD3/CD28 Abs (at a bead/cell ratio of 1:1) at 37°C in a 5% CO_2_ incubator for 3 days. The corresponding T cell culture supernatants were collected to detect cytokine levels in an ELISA assay. Levels of T cell-secreted cytokines, including IFN-γ and IL-10, were analyzed using the ELISA kits (Dakewe Biotech Co., Ltd., Shenzhen, China) as per the manufacturer’s instructions.

### Quantitative Real-time RT-PCR (qRT-PCR) Analysis

Interleukin-6, CD126, gp130, SOCS1, SOCS2, SOCS3, ADAM10, and ADAM17 mRNA expression in MDSCs isolated from primary breast cancer tissues and *in vitro*-induced MDSCs was analyzed by qRT-PCR. The mRNA levels of target genes were quantified using the SYBR Premix Ex Taq TM system (Takara Bio, Shiga, Japan) with an ABI 7500 instrument (Applied Biosystems, Foster City, CA, USA). The primers for IL-6, CD126, gp130, SOCS1–3, ADAM10, ADAM17, and β-actin are shown in Table 2. Relative mRNA levels in each sample were calculated based on their threshold cycle (Ct) values normalized to the Ct value of β-actin using the formula: 2^−ΔCt^ (ΔCt = Ct_target gene_ − Ct_β-actin_). All tests were conducted at least five times.

**Table 2:** The RT-PCR primers of interested genes.

Genes	Primer sequences		Bases
Interleukin-6 (IL-6)	Up	CAATGAGGAGACTTGCCTGG	20
	Down	GGCATTTGTGGTTGGGTCAG	20
SOCS1	Up	GACGCCTGCGGATTCTACT	19
	Down	AGGCCATCTTCACGCTAAGG	20
SOCS2	Up	CGCTATCCTTCCCTGAACC	19
	Down	GTCCGAAATGGTGGCAGA	18
SOCS3	Up	AAGCACAAGAAGCCAACCAG	20
	Down	TTCCCTCCAACACATTCCAG	20
CD126	Up	TTGGACACTCACACGGACA	19
	Down	GAGGCTTTGGCTGGAAATC	19
gp130	Up	ACACCAAGTTCCGTCAGTCC	20
	Down	TACCATCACCGCCATCTACA	20
ADAM10	Up	GCTCATTGGTGGGCAGTATT	20
	Down	GTGGTTTAGGAGGAGGCAACT	21
ADAM17	Up	ACTGCACGTTGAAGGAAGGT	20
	Down	ACGCCTTTGCAAGTAGCATT	20
β-actin	Up	TGGCACCCAGCACAATGAA	19
	Down	CTAAGTCATAGTCCGCCTAGAAGCA	25

*Up, upstream primer; down, downstream primer*.

### Western Blot Analysis

Western blot analysis was performed to detect the levels of CD126, gp130, ADAM10, ADAM17, and SOCS1–3 proteins, as well as total and phosphorylated JAK1, JAK2, TYK2, STAT1, and STAT3 in MDSCs and CD33^+^ control cells. Cell lysates were separated by SDS-PAGE and transferred to polyvinylidene difluoride membranes for western blot analysis using mouse anti-human CD126, gp130 (R&D Systems, Inc., Minneapolis, MN, USA), SOCS1 (Medical & Biological Laboratories Co., Ltd., Nagoya, Japan), SOCS2–3 (R&D Systems, Inc.), and β-actin. Rabbit anti-human antibodies were used to detect JAK1, JAK2, TYK2, STAT1, STAT3, p-STAT1, p-JAK1, p-JAK2, p-TYK2, and p-STAT3 (Cell Signaling Technology, Danvers, MA, USA). Membranes were incubated with primary antibodies overnight at 4°C, as described previously (7). Membranes were then incubated with horseradish peroxidase-conjugated anti-mouse or anti-rabbit IgG Ab (Zhongshanjinqiao, Beijing, China), and protein bands were visualized using the SuperSignal West Pico Chemiluminescent Substrate kit (Pierce Biotechnology, Rockford, IL, USA). The relative densities of protein bands were determined by comparing the band densities of proteins of interest to those of β-actin, using Quantity One software. We used the density ratio of phosphorylated protein to total protein to compare the expression of these phosphorylated proteins.

### Statistical Analysis

Statistical analyses were performed using the SPSS 20.0 (SPSS, Inc., Chicago, IL, USA) and GraphPad Prism 5.0 software (GraphPad, Inc., La Jolla, CA, USA). Measured data were presented as the mean ± SD; one-way analysis of variance and least significant difference tests were used to compare quantitative data. Categorical data were presented as the median, and the nonparametric χ^2^ test was used to compare qualitative data. The cumulative survival probability was determined by the Kaplan–Meier method, and the log-rank test was used to compare overall survival (OS) of each subgroup of patients. *P*-values for each analysis are reported in the figure legends, and the level of statistical significance was set to *P* < 0.05.

## Results

### Tumor-Derived IL-6 and Local MDSCs Infiltration Are Significantly Correlated with Lymph Node Metastasis and Poor Prognosis in Breast Cancer Patients

**Figure 1:**
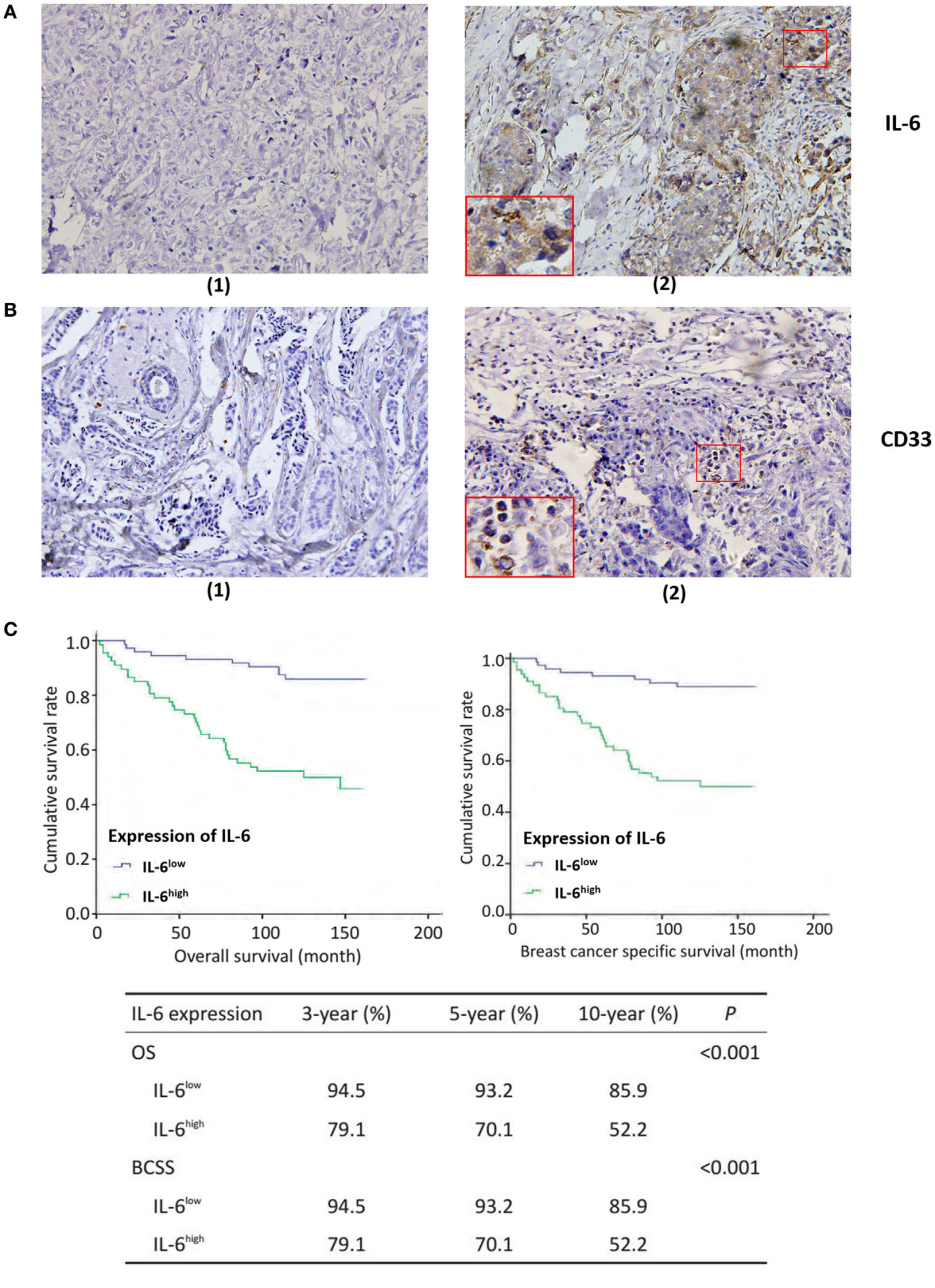

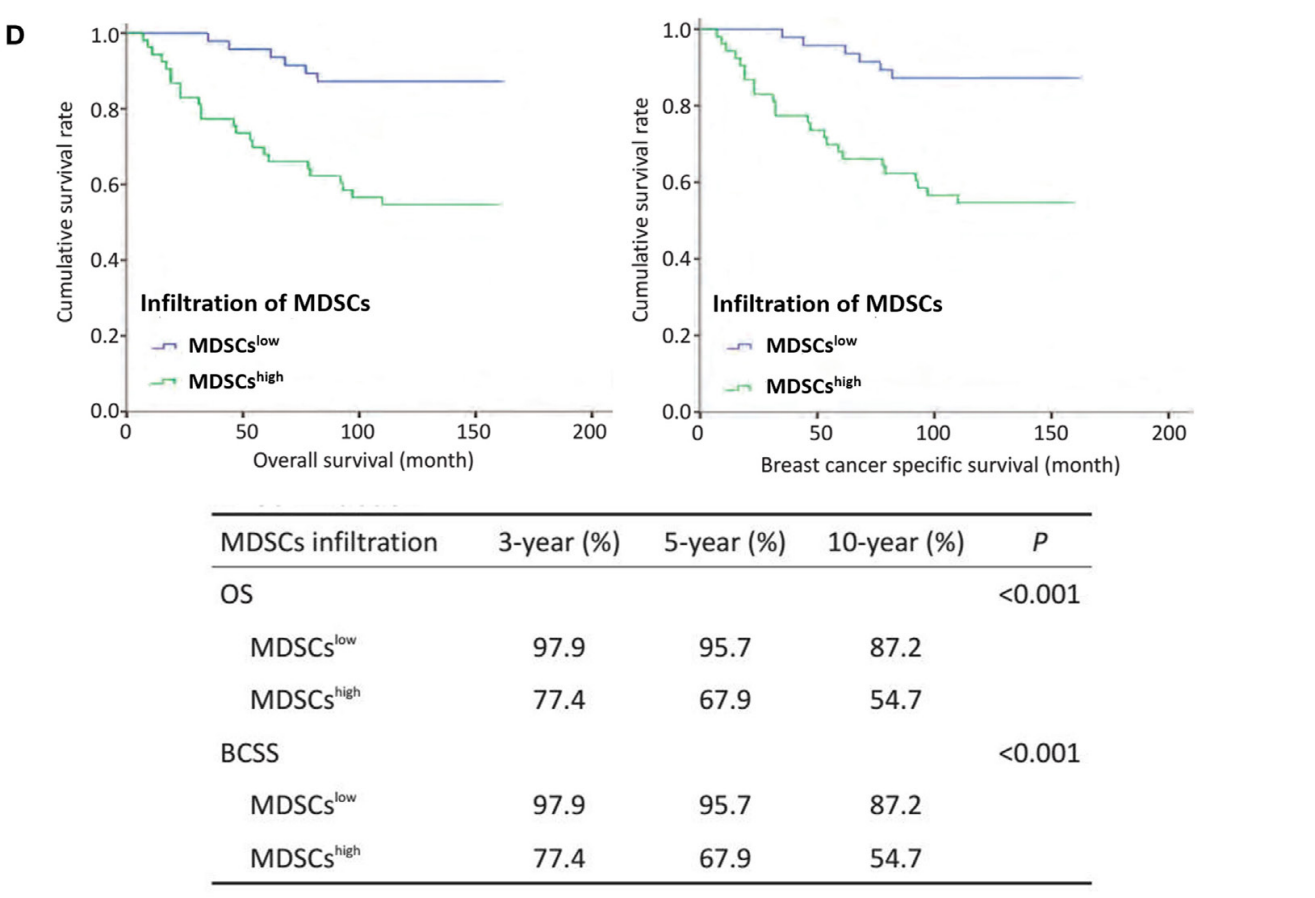
Tumor-derived interleukin-6 (IL-6) and local myeloid-derived suppressor cells (MDSCs) infiltration are significantly correlated with lymph node metastasis and poor prognosis in breast cancer patients. 253 breast cancer patients were selected for immunohistochemistry (IHC) staining of IL-6 and MDSCs, original magnification ×200. Five representative high-power fields (×400) for each tissue section were selected for histology evaluation. **(A)** (1) Low expression of IL-6; (2) high expression of IL-6. IL-6 was mainly expressed in the cytoplasm of breast cancer cells, as well as in some mesenchymal cells. **(B)** (1) Low infiltration of MDSCs; (2) The representative high infiltration of MDSCs. We defined the mesenchymal cells expressing CD33 antigen molecules as MDSCs and CD33^+^ MDSCs were scattered in the stroma of breast cancer tissues with varying sizes and shapes. **(C)** The overall survival (OS) of the 140 patients in cohort 2 with IL-6 expression and MDSCs infiltration was analyzed using Kaplan–Meier and the Log rank test. The OS, BCSS, 3-, 5-, and 10-year survival rate of IL-6^high^ patients were significantly shorter than those of IL-6^low^ patients (*P* < 0.001). **(D)** Similar results were observed in MDSCs^high^ patients as compared with MDSCs^low^ patients (*P* < 0.001).

Interleukin-6 was mainly expressed in the cytoplasm of breast cancer cells, as well as in some mesenchymal cells (Figure 1A). Based on the staining intensity and extent of IL-6 expression, breast cancer patients were divided into an IL-6 low expression group (IL-6^low^) and high expression group (IL-6^high^). The IL-6^high^ cases accounted for 44.4% (48/108) of patients in cohort 1 and 50.5% (50/99) of patients in cohort 2. CD33^+^ MDSCs were scattered in the stroma of breast cancer tissues with varying sizes and shapes (Figure 1B). According to the number of CD33^+^ MDSCs that infiltrated locally, breast cancer patients were divided into lowly infiltrated MDSC group (MDSCs^low^) and highly infiltrated MDSC group (MDSCs^high^). The MDSCs^high^ cases accounted for 52.0% of patients in cohort 1 and 50.9% of patients in cohort 2.

We next compared the correlations between IL-6 expression, MDSCs infiltration, other clinical pathological features (age, agenda, tumor size, tumor pathologic stage, tumor histological grade, and lymph node invasion), and expression of hormone receptors (estrogen receptor, progesterone receptor, and HER2) in the two cohorts. As shown in Table 3, tumor-derived IL-6 expression was significantly correlated with lymph node invasion and tumor histological grade; compared to IL-6^low^ patients, IL-6^high^ patients suffered from more aggressive histological features (cohort 1: *P* < 0.001; cohort 2: *P* = 0.001) and a higher risk of early lymph node invasion (cohort 1: *P* < 0.001; cohort 2: *P* = 0.012). Similar trends were observed in MDSC^high^ patients as compared with MDSC^low^ patients, where more infiltrated MDSCs were detected in cancer tissues at more advanced pathological stages, with higher histological grade, more lymph node metastasis, and larger tumor size (*P* < 0.001, *P* < 0.001, *P* = 0.025, *P* = 0.018; *P* < 0.001, *P* = 0.007; *P* = 0.022, *P* = 0.032, respectively, Table 4). These results demonstrated that breast cancers with higher IL-6 expression and greater MDSC infiltration possess a higher potential for invasion and metastasis.

**Table 3:** Correlations of interleukin-6 (IL-6) with clinicopathological features of breast cancer patients.

Baseline	Cohort 1			Cohort 2		
	IL-6^low^	IL-6^high^	*P*	IL-6^low^	IL-6^high^	*P*
Age			0.870			0.284
≤52 years	33	24		37	40	
>52 years	31	24		36	27	
Histology grade		0.208			0.523
0–I	7	2		11	8	
II–III	55	43		59	59	
Lymph node			<0.001			0.012
Negative	58	23		51	33	
Positive	6	22		20	32	
Tumor size			0.060			0.936
≤3 cm	50	29		42	39	
>3 cm	13	17		31	28	
Stage			<0.001			0.001
0–II	59	24		56	35	
III–IV	5	24		15	32	
ER			0.986			0.059
Negative	21	16		21	30	
Positive	43	33		47	34	
PR			0.256			0.232
Negative	22	22		28	33	
Positive	42	27		40	31	
HER2			0.242			0.681
Negative	38	24		50	45	
Positive	14	15		18	19	

**Table 4:** Correlations of myeloid-derived suppressor cells (MDSCs) with the clinicopathological characteristics of breast cancer patients.

Baseline	Cohort 1			Cohort 2		
	MDSCs^low^	MDSCs^high^	*P*	MDSCs^low^	MDSCs^high^	*P*
**Age**			0.503			0.952
≤52 years	25	30		26	29	
>52 years	27	25		21	24	
**Histology grade**		0.025			0.018
0–I	7	1		10	3	
II–III	44	51		36	50	
**Lymph node**		<0.001			0.007
Negative	50	28		34	26	
Positive	2	24		11	27	
**Tumor size**			0.022			0.032
≤3 cm	42	33		33	26	
>3 cm	9	20		14	27	
**Stage**			<0.001			<0.001
0–II	51	28		40	26	
III–IV	1	27		5	27	
**ER**			0.586			0.074
Negative	16	20		14	25	
Positive	36	36		30	25	
**PR**			0.287			0.215
Negative	18	25		19	28	
Positive	34	31		25	22	
**HER2**			0.404			0.177
Negative	29	28		33	31	
Positive	12	17		11	19	

Next, we compared the OS of the 140 patients in cohort 2 with IL-6 expression and MDSC infiltration. We found that the OS, 3-, 5-, and 10-year survival of IL-6^high^ patients, was significantly shorter than those of IL-6^low^ patients (*P* < 0.001, Figure 1C). Similarly, IL-6^high^ patients displayed worse overall breast cancer-specific survival, 3-, 5-, and 10-year breast cancer-specific survival compared to IL-6^low^ patients (*P* < 0.001, Figure 1C). Similar results were observed in MDSC^high^ patients compared to MDSC^low^ patients (*P* < 0.001, Figure 1D). Thus, these findings indicate that tumor-derived IL-6 and MDSC infiltration are both unfavorable prognostic factors in breast cancer and are significantly correlated with aggressive tumor behavior and poor clinical outcomes in patients.

### Tumor-Derived IL-6 Is Significantly Correlated with the Number of Infiltrated MDSCs *In Situ* Both at the mRNA and Protein Levels

**Figure 2:**
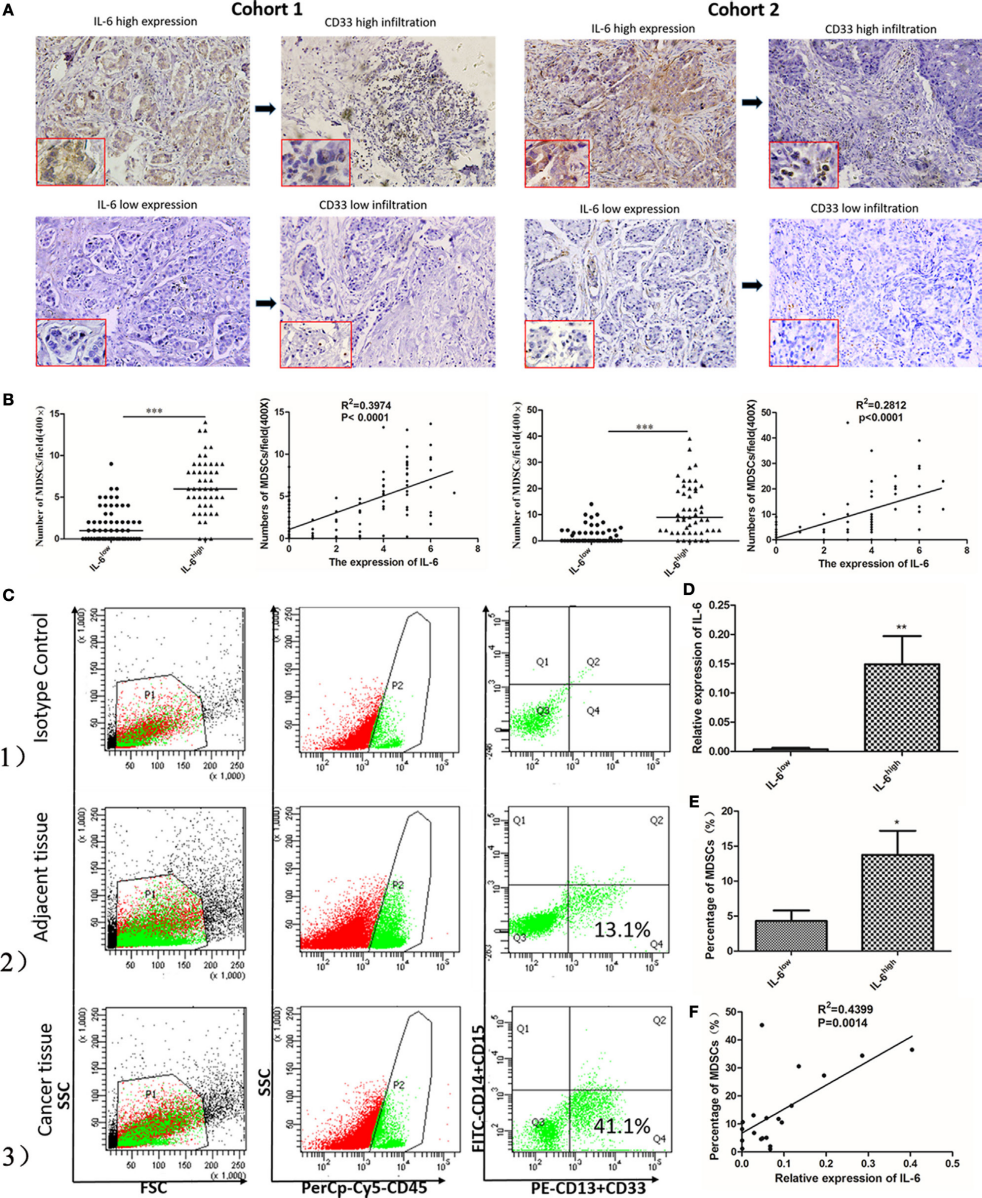
Tumor-derived interleukin-6 (IL-6) is significantly correlated with the number of infiltrated myeloid-derived suppressor cells (MDSCs) *in situ* both at the mRNA and protein levels. **(A)** The expression of the IL-6 protein and CD33^+^ MDSCs infiltration in 253 paraffin-embedded breast tissues from cohort 1 and cohort 2 was studied by immunohistochemistry (IHC). We found greater MDSCs infiltration in cancer tissues with a high level of IL-6. **(B)** The correlation between the expression of IL-6 and MDSCs was compared both in cohort 1 and cohort 2 *in situ* (*n* = 253). The average number of MDSCs in the IL-6^low^ group was significantly lower than that in the IL-6^high^ group in both cohorts 1 and 2. Pearson correlation analysis revealed a positive correlation between the expression of IL-6 and the number of MDSCs *in situ* in both cohorts. **(C)** The infiltration percentage of the CD45^+^CD33^+^CD13^+^CD14^−^CD15^−^ subpopulation in 20 fresh breast cancer tissue samples was detected using flow cytometry. (1) The subpopulation was gated using anti-CD45 mAb and isotype control was used; (2) The CD45^+^CD33^+^CD13^+^CD14^−^CD15^−^ subpopulation in adjacent normal tissues. (3) The proportion of the interested subpopulation significantly increased in cancer tissues. **(D)** Based on the median relative RNA level of IL-6, breast cancer samples were divided into IL-6^high^ and IL-6^low^ groups. The average IL-6 mRNA level in the IL-6^high^ group was 37.25-fold higher than that in the IL-6^low^ group (*P* = 0.0093) (*n* = 20). **(E)** A higher frequency of MDSCs was detected in the IL-6^high^ group compared to in the IL-6^low^ group. **(F)** A correlation analysis on MDSCs number and IL-6 level was carried out (*R*^2^ = 0.4399, *P* = 0.0014) (*n* = 20). *P < 0.05, **P < 0.01, ***P < 0.001.

We compared the correlation between the expression of IL-6 and number of infiltrated MDSCs *in situ* to evaluate the effects of IL-6 on MDSC accumulation in breast cancer tissues. We first studied the expression of IL-6 protein in 253 paraffin-embedded breast tissues from cohorts 1 and 2 by IHC. We found greater MDSC infiltration in cancer tissues with a high level of IL-6 (Figure 2A). The average number of MDSCs in the IL-6^low^ group was significantly lower than that in the IL-6^high^ group in both cohorts 1 and 2 [(1.95 ± 0.26) vs. (6.40 ± 0.48), *P* < 0.001; (1.31 ± 0.27) vs. (6.43 ± 0.79), *P* < 0.001, Figure 2B]. Pearson correlation analysis revealed a positive correlation between the expression of IL-6 and the number of MDSCs *in situ* in both cohorts (cohort 1, *R*^2^ = 0.3974, *P* < 0.0001; cohort 2, *R*^2^ = 0.2812, *P* < 0.0001, Figure 2B).

Twenty fresh breast cancer tissue samples were collected to study the correlation between RNA levels of tumor-derived IL-6 and percentages of CD45^+^CD33^+^CD13^+^CD14^−^CD15^−^ MDSCs in breast cancer tissues by flow cytometry analysis. We observed a cluster of CD33^+^CD13^+^CD14^−^CD15^−^ cells in breast cancer tissue, which represented the predominant phenotype of MDSCs in breast cancer (Figure 2C). The percentage of CD45^+^CD33^+^CD13^+^CD14^−^CD15^−^ MDSCs was 15.3–58.1% with a mean value of 29.82 ± 11.463%. Based on the median relative RNA level of IL-6, breast cancer samples were divided into IL-6^high^ and IL-6^low^ groups. The average IL-6 mRNA level in the IL-6^high^ group was 37.25-fold higher than that in the IL-6^low^ group (*P* = 0.0093, Figure 2D). Higher frequency of MDSCs was detected in the IL-6^high^ group compared to in the IL-6^low^ group [(13.75 ± 3.44%) vs. (4.31 ± 1.50%), *P* = 0.03, Figure 2E]. Furthermore, Pearson correlation analysis revealed a strong positive correlation between the expression of IL-6 mRNA and number of MDSCs in fresh breast cancer tissues (*R*^2^ = 0.4399, *P* = 0.0014, Figure 2F).

### IL-6 Enhanced the Generation and T Cell Immunosuppressive Ability of MDSCs *In Vitro*

**Figure 3:**
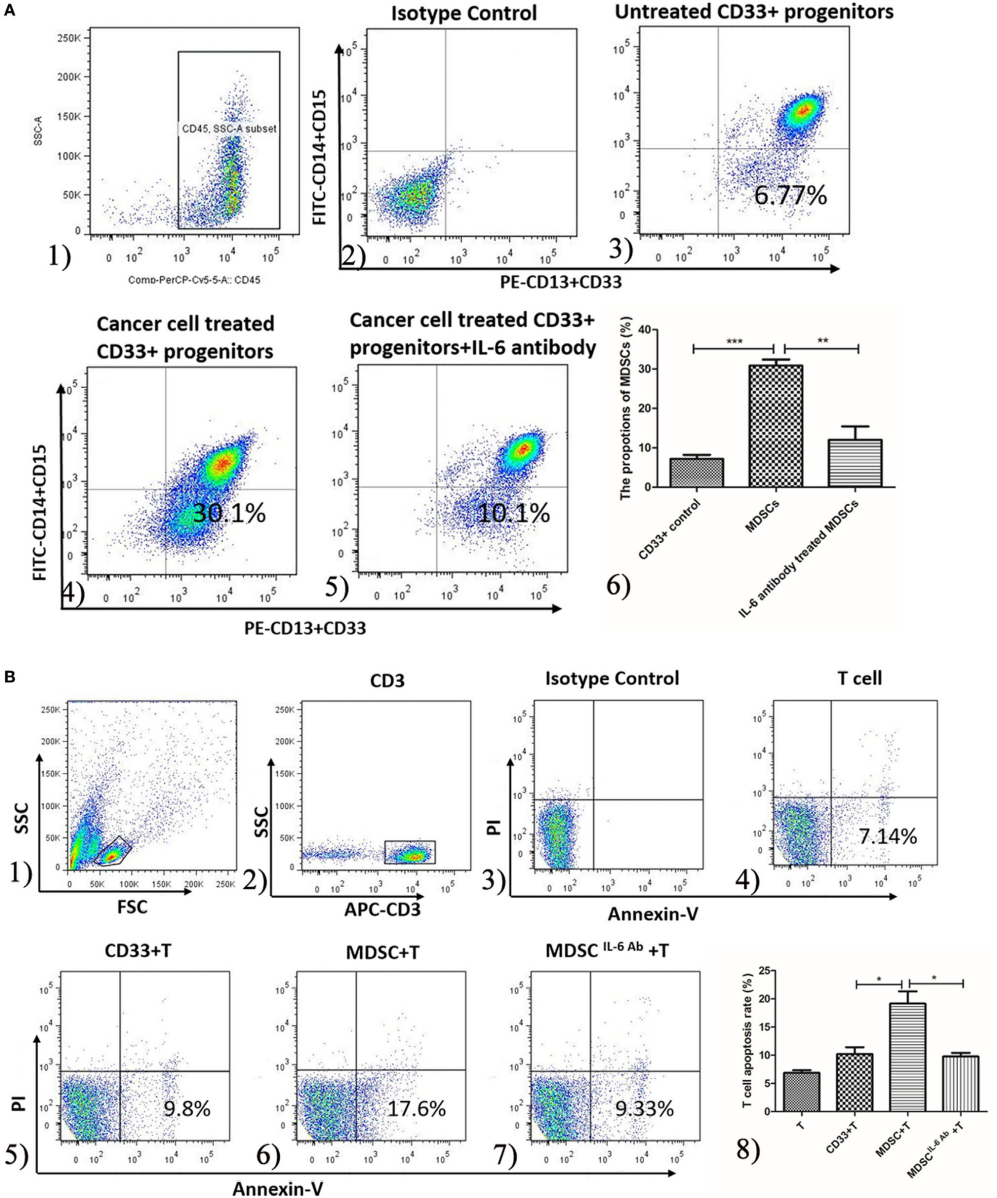

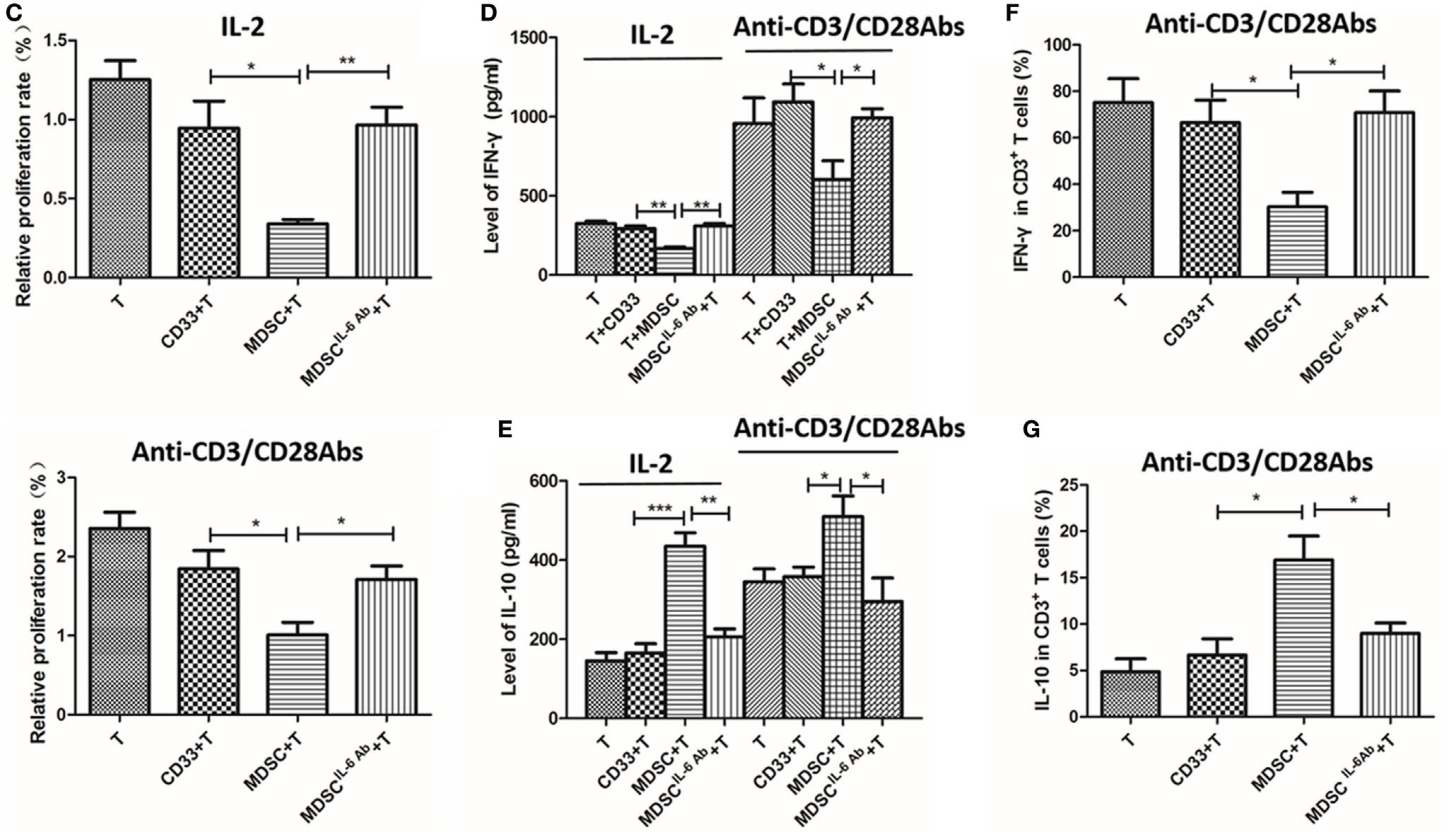
Interleukin-6 (IL-6) enhanced the generation and T cells immunosuppressive ability of myeloid-derived suppressor cells (MDSCs) *in vitro*. **(A)** The proportion of healthy people’s untreated CD33^+^ myeloid progenitors and the treated CD33^+^ cells was compared using flow cytometry method. Cells in Q4 represent MDSCs. (1) The subpopulation was gated using anti-CD45 mAb; (2) isotype control was used; (3) the proportion range of CD33^+^ was 15.6 ± 2.6%; (4) when treated with cancer cells the proportion of MDSCs was highly increased which was 30.83 ± 1.595%; (5) an IL-6 neutralizing antibody was added to the cancer-conditioned MDSCs culture and MDSCs decreased to 11.98 ± 3.479%; (6) the result of statistical analysis (*n* = 6). The effects of MDSCs and CD33^+^ cells on T cell proliferation, apoptosis and cytokine secretion were examined **(B–E)**. **(B)** T cells stimulated with 1,000 IU/ml IL-2 were co-cultured with CD33^+^ control cells or MDSCs at ratio of 1:3 for detecting apoptosis. T cells were gated using APC-labeled anti-CD3 mAb, and apoptotic cells were stained with FITC-labeled Annexin V. Cells in Q4 represent apoptotic T cells. (1) The lymphocytes were gated according to SSC and FSC features; (2) CD3 mAb labeled T cells; (3) isotype control; (4) T cells only; (5) CD33^+^ controls stimulated T cells; (6) MDSCs stimulated T cells; (7) T cells were co-cultured with MDSCs in the presence of IL-6 antibody; (8) Summary of (4–7) MDSCs stimulated greater T cell apoptosis compared to in CD33^+^ controls. In addition, the IL-6 antibody dramatically abolished MDSCs-induced T cell apoptosis (*n* = 5). **(C)** The proliferation of T cells was detected using CCK8 method. Compared to in CD33^+^ controls, MDSCs significantly inhibited IL-2 or anti-CD3/CD28 Abs-induced proliferation of T cells at a ratio of 1:3, which was attenuated by IL-6 blocking antibody (*n* = 5). **(D,E)** Supernatants were collected for detecting interferon (IFN)-γ and IL-10 level using ELISA assay (*n* = 5). **(D)** MDSCs inhibited IL-2 or anti-CD3/CD28 Abs-induced IFN-γ secretion, which was increased after IL-6 antibody pretreatment. **(E)** In contrast, MDSCs stimulated IL-10 secretion in IL-2 or anti-CD3/CD28 Abs simulated T cells compared to in CD33^+^ controls, which was dropped significantly after IL-6 blocking. **(F)** Flow cytometry was used to detect IFN-γ expression by intracellular staining. The proportion of IFN-γ-positive T cells decreased after co-culture with MDSCs compared to in CD33^+^ controls, but MDSCs-mediated inhibition of IFN-γ production in T cells was reversed by blocking of IL-6 (*n* = 5). **(G)** In contrast, the percentages of IL-10-positive T cells increased after co-culture with MDSCs and blocking of IL-6 inhibited IL-10 production in T cells (*n* = 5). *P < 0.05, **P < 0.01, ***P < 0.001.

To mimic the breast cancer cell-conditioned microenvironment *in vitro*, CD33^+^ myeloid progenitors were isolated from healthy donors’ PMBCs and co-cultured with MDA-MB-231 breast cancer cells. After 48 h of culture, the proportion of MDSCs possessing the CD45^+^CD33^+^CD13^+^CD14^−^CD15^−^ phenotype was increased from 15.6 ± 2.6 to 30.83 ± 1.595% (*P* = 0.015; Figure 3A).

To determine whether IL-6 plays a major role in promoting MDSC differentiation *in vitro*, an IL-6 neutralizing antibody was added to the cancer-conditioned MDSC culture. The proportion of CD45^+^CD33^+^CD13^+^CD14^−^CD15^−^ cells in IL-6-neutralizing antibody-treated MDSCs (Ab-treated MDSCs) was dramatically decreased compared to in untreated MDSCs (Ab-untreated MDSCs) [(11.98 ± 3.479%) vs. (30.83 ± 1.595%), *P* = 0.0007, Figure 3A]. These results indicate that breast cancer-induced IL-6 secretion significantly promotes the differentiation and accumulation of MDSCs *in vitro*.

To examine whether IL-6 regulates MDSCs-mediated immunosuppressive effects on T cells *in vitro*, we co-cultured both Ab-treated MDSCs and Ab-untreated MDSCs with T cells isolated from exogenous PMBCs and examined the proliferation, apoptosis, and cytokine production of T cells. MDSCs stimulated more apoptotic T cell compared to CD33^+^ controls [(19.17 ± 2.12%) vs. (10.28 ± 1.26%), *P* = 0.0240, Figure 3B]. In addition, the IL-6 antibody dramatically abolished MDSCs-induced T cell apoptosis [(9.797 ± 0.6411%) vs. (19.20 ± 2.13%), *P* = 0.0151, Figure 3B]. Accordingly, compared to CD33^+^ controls, MDSCs significantly inhibited IL-2-induced proliferation of T cells at a ratio of 1:3 (0.9452 ± 0.1721 vs. 0.3410 ± 0.02694, *P* = 0.0256, Figure 3C). The IL-6-blocking antibody fully reversed MDSCs-mediated inhibition on T cell proliferation (0.9655 ± 0.1131, *P* = 0.0058, Figure 3C). Similarly, anti-CD3/CD28 Abs-induced T cell proliferation was significantly inhibited by MDSCs (*P* = 0.0416), and the IL-6-blocking antibody reversed MDSCs-mediated inhibition on anti-CD3/CD28 Abs-induced T cell proliferation (*P* = 0.0404, Figure 3C).

Finally, we evaluated whether IL-6 modulates MDSCs-mediated suppression of cytokine secretion in T cells. IFN-γ secretion in IL-2-stimulated T cells was inhibited by MDSCs from 293.7 ± 17.47 to 168.6 ± 9.498 pg/mL (*P* < 0.01, Figure 3D). However, IL-6 blocking antibody eliminated MDSCs-mediated suppression on IFN-γ secretion, which increased to 310.0 ± 15.57 pg/mL (*P* = 0.0015, Figure 3D) after IL-6 antibody pretreatment. Consistently, anti-CD3/CD28 Abs-induced IFN-γ secretion of T cells was suppressed (1,094 ± 113.4 vs. 602.0 ± 120.5 pg/mL, *P* = 0.0410, Figure 3D) by MDSCs, but after IL-6 antibody pretreatment, the secretion of IFN-γ increased (992.8 ± 57.90 pg/mL, *P* = 0.0238, Figure 3D). In contrast, MDSCs stimulated more IL-10 secretion in IL-2-simulated T cells than CD33^+^ controls, which increased from 434.8 ± 34.52 to 165.4 ± 23.39 pg/mL (*P* < 0.001, Figure 3E). However, IL-6-blocking antibody eliminated MDSCs-mediated increase of IL-10, which significantly decreased to 205.7 ± 20.54 pg/mL (*P* = 0.0013, Figure 3E). Consistently, IL-10 secretion in anti-CD3/CD28 Abs-stimulated T cells was promoted by MDSCs compared to CD33^+^ controls (345.4 ± 35.68 vs. 509.8 ± 52.25 pg/mL, *P* = 0.0386, Figure 3E) and IL-6-blocking antibody eliminated MDSCs-mediated effect on IL-10 secretion (295.4 ± 59.25 pg/mL, *P* = 0.0349, Figure 3E).

Furthermore, we detected IFN-γ and IL-10 production in anti-CD3/CD28 Abs-stimulated T cells using an intracellular staining method. The results showed that the proportion of IFN-γ-positive T cells decreased after co-culture with MDSCs compared to CD33^+^ controls (72.40 ± 5.771 vs. 42.40 ± 8.965%, *P* = 0.0481, Figure 3F), but MDSCs-mediated inhibition of IFN-γ production in T cells was reversed by blocking IL-6 (73.20 ± 4.574%, *P* = 0.0376, Figure 3F). In contrast, the percentages of IL-10-positive T cells increased after co-culture with MDSCs (from 6.650 ± 1.751 to 16.91 ± 2.570%, *P* = 0.0299, Figure 3G) and blocking IL-6 inhibited IL-10 production in T cells (8.990 ± 1.123%, *P* = 0.0476, Figure 3G). These results indicate that MDSCs-induced immunosuppressive effects on T cells were IL-6-dependent and could be fully attenuated by blocking IL-6 signaling.

### IL-6 Stimulated Sustained Activation of the JAK/STAT Pathway in MDSCs Displaying Persistent Phosphorylation of Downstream STAT Proteins

**Figure 4:**
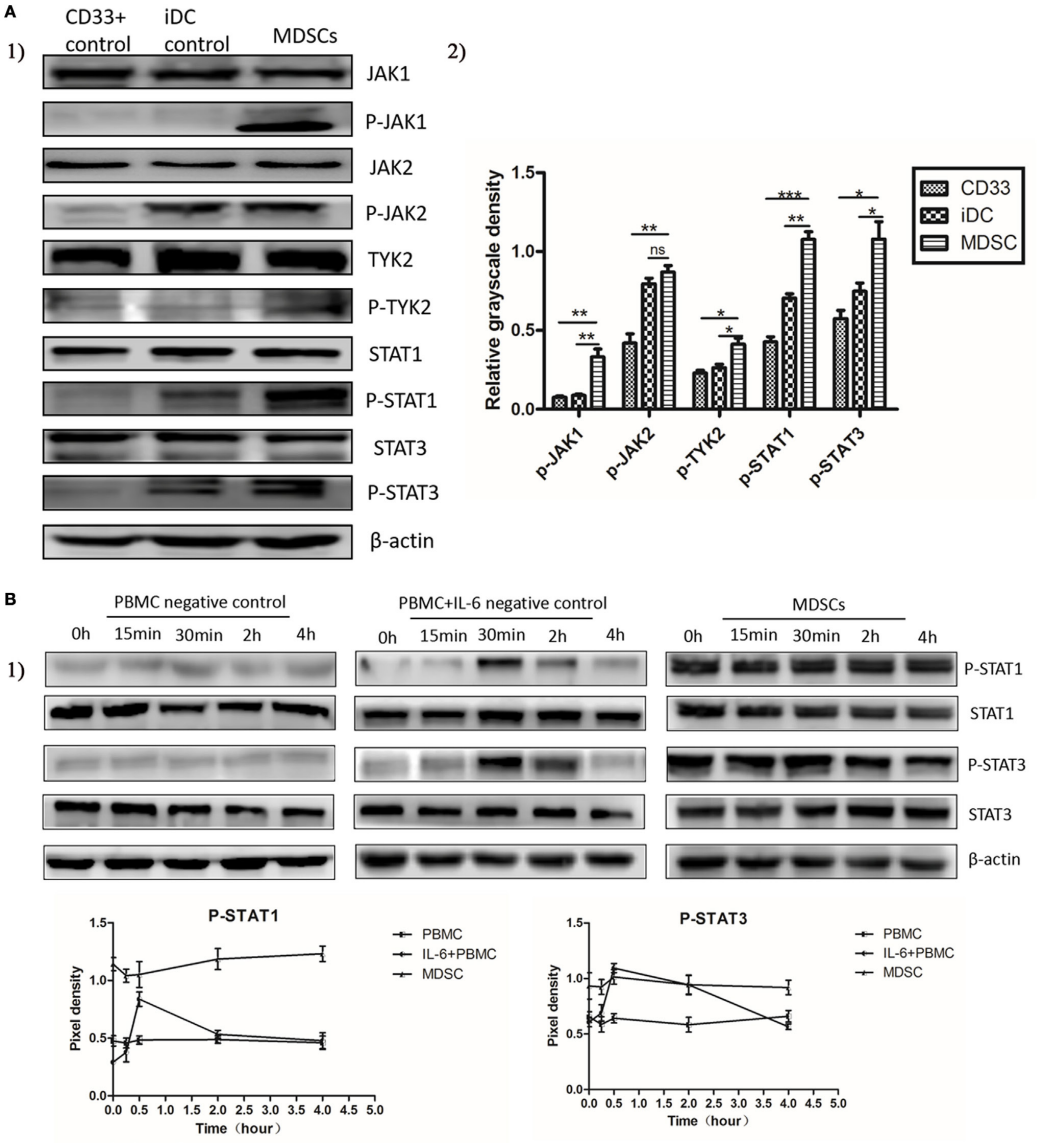

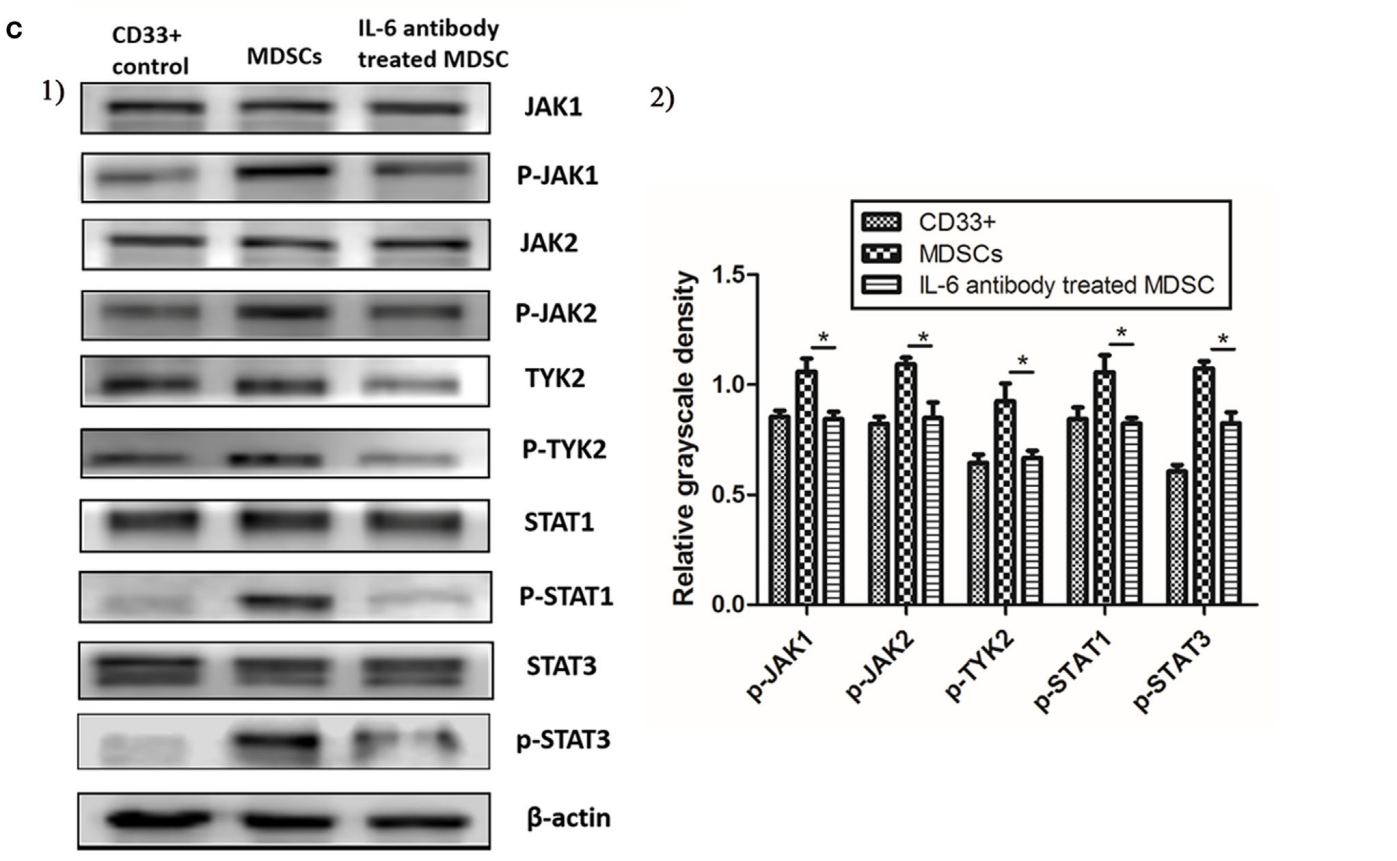
Interleukin-6 (IL-6) stimulated the sustained activation of the JAK/STAT pathway in myeloid-derived suppressor cells (MDSCs) displaying persistent phosphorylation of downstream STAT proteins. The activation status of the JAK/STAT pathway was measured using Western blot. **(A)** Comparable increases in phosphorylated JAK1, JAK2, TYK2, STAT1, and STAT3 proteins were detected in MDSCs as compared to in CD33^+^ controls (*n* = 3). **(B)** Furthermore, sustained phosphorylation of STAT1 and STAT3 proteins was observed in MDSCs, which was maintained for a longer time than in normal IL-6-stimulated PBMCs. In IL-6 (100 ng/mL)-stimulated PBMCs, the levels of phosphorylated STAT1 and STAT3 proteins were increased at 30 min, but decreased at 2 h, disappearing entirely at 4 h. In contrast, persistent IL-6-induced STAT1 and STAT3 phosphorylation in MDSCs lasted for more than 4 h (*n* = 3). **(C)** In contrast, persistent IL-6-induced STAT1 and STAT3 phosphorylation in MDSCs lasted for more than 4 h. After adding an IL-6-blocking antibody, phosphorylation levels of the above proteins were reduced significantly in MDSCs. They were compared using the density ratio of phosphorylated protein to total protein (*n* = 3). *P < 0.05, **P < 0.01, ***P < 0.001.

To elucidate the molecular mechanisms regulating IL-6-induced MDSC differentiation and immunosuppressive activities, we studied the activation status of the JAK/STAT pathway downstream of IL-6 signaling. We assessed the expression and phosphorylation of multiple functional proteins along the JAK/STAT pathway, such as JAK1, JAK2, TYK2, STAT1, and STAT3, using western blot assays. Comparable increases in phosphorylated JAK1, JAK2, TYK2, STAT1, and STAT3 proteins were detected in MDSCs as compared to that in CD33^+^ controls (Figure 4A). Furthermore, sustained phosphorylation of STAT1 and STAT3 proteins was observed in MDSCs, which was maintained for a longer time than in normal IL-6-stimulated PBMCs (2 vs. 4 h, Figure 4B). In IL-6 (100 ng/mL)-stimulated PBMCs, the levels of phosphorylated STAT1 and STAT3 proteins were increased at 30 min, but decreased at 2 h, disappearing entirely at 4 h (Figure 4B). In contrast, persistent IL-6-induced STAT1 and STAT3 phosphorylation in MDSCs lasted for more than 4 h. After adding an IL-6 blocking antibody, phosphorylation levels of the above proteins were reduced significantly in MDSCs, including p-JAK1 (1.059 ± 0.06000 vs. 0.8431 ± 0.03423, *P* = 0.0354), p-JAK2 (1.093 ± 0.03076 vs. 0.8486 ± 0.07076, *P* = 0.0340), p-TYK2 (0.9248 ± 0.08132 vs. 0.6939 ± 0.01329, *P* = 0.0487), p-STAT1 (1.056 ± 0.07766 vs. 0.8229 ± 0.02599, *P* = 0.0464), and p-STAT3 (1.074 ± 0.03318 vs. 0.8247 ± 0.04921, *P* = 0.0137, Figure 4C). These data indicate that enhanced phosphorylation of STAT proteins in MDSCs is IL-6-dependent, although additional factors along the JAK/STAT pathway can manipulate persistent IL-6-induced activation of both STAT1 and STAT3 proteins.

### IL-6-Induced Suppression of SOCS3 in MDSCs Was Determined at both the mRNA and Protein Levels

**Figure 5:**
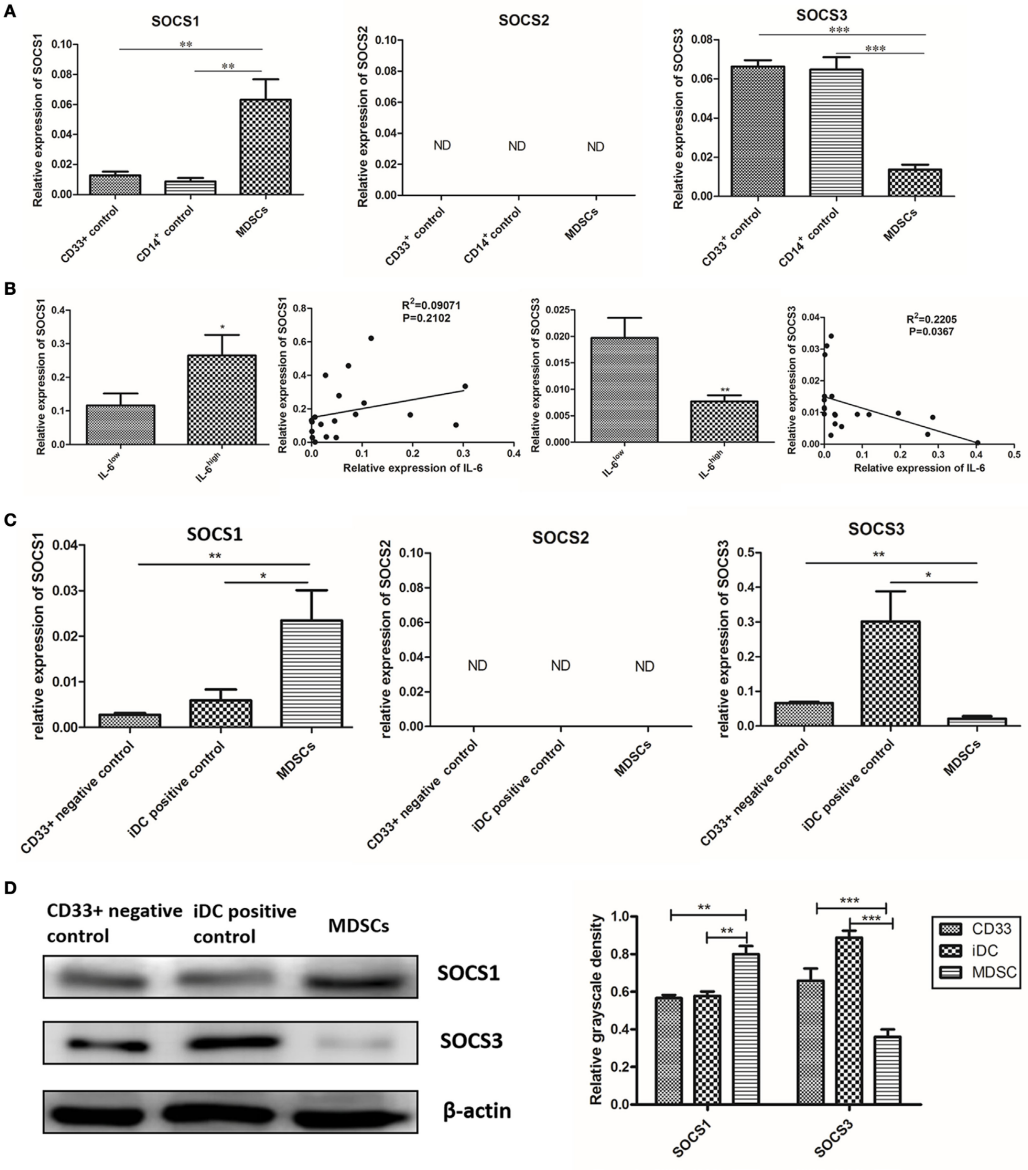

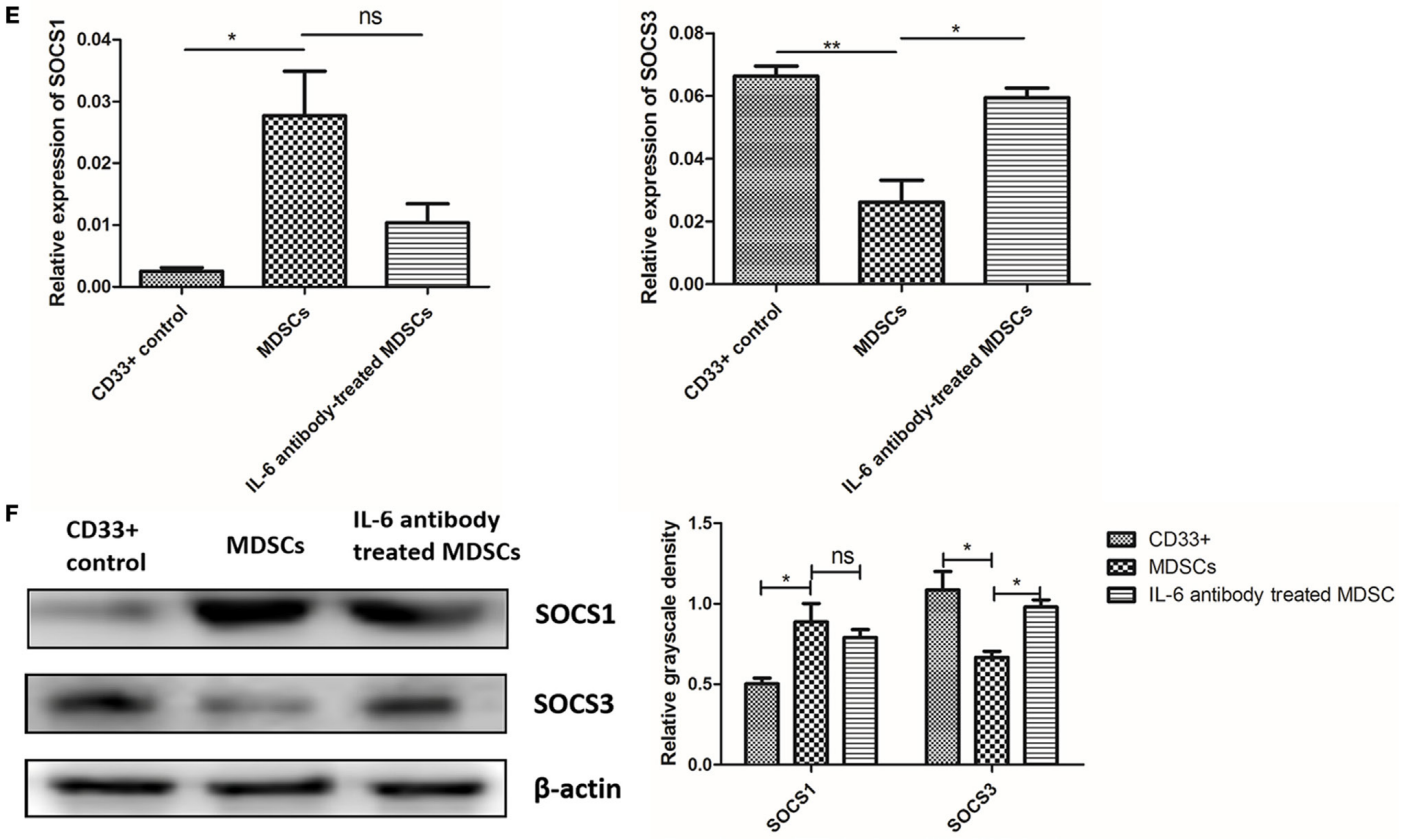
Interleukin-6 (IL-6)-induced suppression of suppressor of cytokine signaling 3 (SOCS3) in myeloid-derived suppressor cells (MDSCs) was determined at both the mRNA and protein levels. **(A)** RT-PCR result showed mRNA level of SOCS1 was increased and SOCS3 was significantly decreased in MDSCs compared to in both CD33^+^ and CD14^+^ controls; an undetectable level of SOCS2 mRNA was observed in both MDSCs and controls (*n* = 20). **(B)** The mRNA levels of SOCS1–3 between MDSCs from IL-6^high^ tissues (MDSC^IL-6h^) and MDSCs from IL-6^low^ tissues (MDSC^IL-6l^) were compared. The mRNA level of SOCS1 was increased in MDSC^IL-6h^ (*P* = 0.0459), while the mRNA level of SOCS3 was decreased in MDSC^IL-6h^ compared to in MDSC^IL-6l^ (*P* = 0.0089). Linear regression analysis demonstrated that IL-6 expression was not correlated with SOCS1 (*R*^2^ = 0.09071, *P* = 0.2102), but demonstrated a significant negative correlation with SOCS3 expression (*R*^2^ = 0.2205, *P* = 0.0367) (*n* = 20). **(C)** Then, we detected mRNA levels of SOCS1–3 in induced MDSCs *in vitro*. The results were consistent with those observed in primary MDSCs, where the mRNA level of SOCS1 was increased, while that of SOCS3 was decreased in MDSCs compared to in the controls (*n* = 5). **(D)** The whole cellular lysates were collected for western blot analysis. SOCS1 protein was notably increased, but SOCS3 protein was dramatically decreased in MDSCs as compared with those in CD33^+^ negative controls and iDC positive controls (*n* = 5). **(E)** RT-PCR show that Ab-treated MDSCs displayed slightly decreased mRNA level in SOCS1 (*P* = 0.0917), but significantly higher mRNA levels in SOCS3 compared to in Ab-untreated MDSCs (*P* = 0.0117) (*n* = 5). **(F)** Consistent results were confirmed at the protein level (*n* = 5). *P < 0.05, **P < 0.01, ***P < 0.001.

Because the loss of SOCS proteins has been reported to induce continuous activation of the JAK/STAT pathway in malignancy, we compared the expression of SOCS1, SOCS2, and SOCS3 between MDSCs and normal myeloid controls at both the mRNA and protein levels. Primary MDSCs isolated from 20 cases of primary breast cancer tissues were studied. CD33^+^ and CD14^+^ cells from healthy donors were regarded as normal myeloid-derived cell controls. We first detected the mRNA levels of SOCS1–3 and found that mRNA level of SOCS1 increased in MDSCs compared to in both CD33^+^ (*P* = 0.006) and CD14^+^ (*P* = 0.003) controls; the mRNA level of SOCS3 significantly decreased in MDSCs compared to in CD33^+^ (*P* < 0.001) and CD14^+^ (*P* < 0.001, Figure 5A) controls. An undetectable level of SOCS2 mRNA was observed in both MDSCs and controls (Figure 5A). We then compared the mRNA levels of SOCS1–3 between MDSCs from IL-6^high^ tissues (MDSC^IL-6h^) and MDSCs from IL-6^low^ tissues (MDSC^IL-6l^). The results demonstrated that the mRNA level of SOCS1 increased in MDSC^IL-6h^ (*P* = 0.0459), while the mRNA level of SOCS3 decreased in MDSC^IL-6h^ compared to that in MDSC^IL-6l^ (*P* = 0.0089, Figure 5B). Linear regression analysis demonstrated that IL-6 expression was not correlated with SOCS1 (*R*^2^ = 0.09071, *P* = 0.2102) but was negatively correlated with SOCS3 expression (*R*^2^ = 0.2205, *P* = 0.0367, Figure 5B).

We then detected the mRNA levels of SOCS1–3 in induced MDSCs *in vitro*. Untreated CD33^+^ myeloid progenitors were used as negative controls, while CD14^+^ monocyte-derived immature DCs (iDC) were used as positive controls. The results were consistent with those observed in primary MDSCs, where the mRNA level of SOCS1 increased, while that of SOCS3 decreased in induced MDSCs (Figure 5C). The disparity between SOCS1 and SOCS3 expression was confirmed at the protein level. The expression of SOCS1 protein notably increased, while that of SOCS3 protein dramatically decreased in MDSCs compared to CD33^+^ negative controls and iDC positive controls (Figure 5D).

To further verify the effect of IL-6 on SOCS3 expression, an IL-6-neutralizing antibody was added to block IL-6 signaling in MDSCs, and the synthesis and expression of SOCS was detected. Ab-treated MDSCs displayed slightly lower mRNA level of SOCS1 (*P* = 0.0917, Figure 5E), but significantly higher mRNA level of SOCS3 compared to that in Ab-untreated MDSCs (*P* = 0.0117, Figure 5E). Consistent results were confirmed at the protein level (Figure 5F). Therefore, our study suggests that IL-6 induces inhibition of SOCS3 expression in MDSCs at both the mRNA and protein levels *in vivo* and *vitro*.

### IL-6-Dependent SOCS3 Suppression and Sustained Activation of the JAK/STAT Pathway Was Correlated with CD126 Upregulation

**Figure 6:**
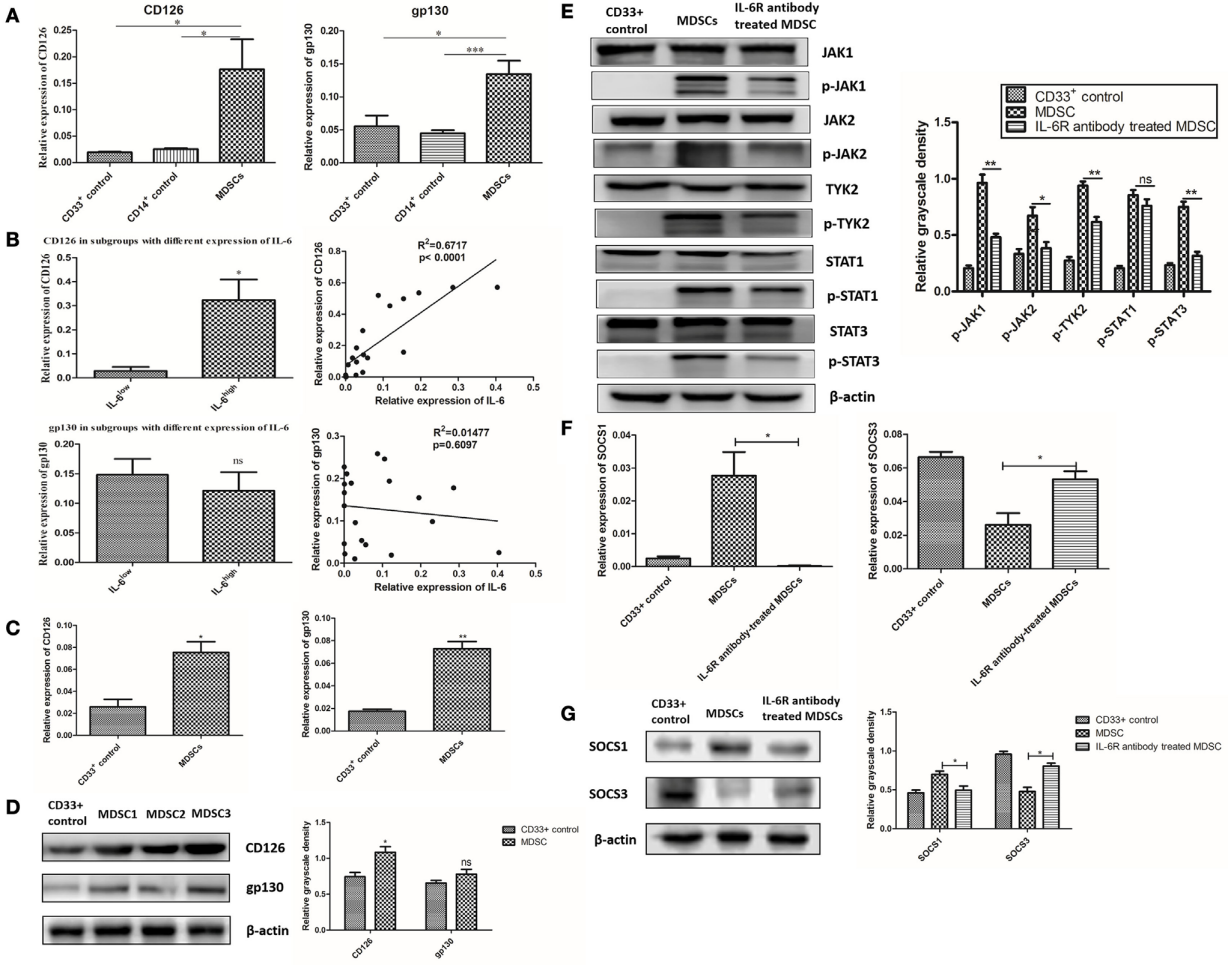
Interleukin-6 (IL-6)-dependent suppressor of cytokine signaling 3 (SOCS3) suppression and sustained activation of the JAK/STAT pathway was correlated with CD126 upregulation. **(A)** The relative expression of interest genes were indicated by 2^−ΔCt^(ΔCt = Ct_target gene_ − Ct_β-actin_). We found the mRNA levels of CD126 and gp130 in primary myeloid-derived suppressor cells (MDSCs) were higher than those in CD33^+^ and CD14^+^ controls (*n* = 5). **(B)** The mRNA levels of CD126 and gp130 in primary MDSCs^IL-6h^ were higher than those in MDSCs^IL-6l^. Linear regression analysis demonstrated that CD126, rather than gp130, was positively correlated with IL-6 levels (*R*^2^ = 0.6717, *P* < 0.0001) (*n* = 20). **(C)** The expressions of CD126 and gp130 in induced MDSCs were also examined. MDSCs exhibited higher mRNA levels of CD126 and gp130 compared to in CD33^+^ controls (*n* = 3). **(D)** Similar results were obtained at the protein level, in which CD126 expression was significantly increased, while gp130 showed no significant changes in the expression (*n* = 3). **(E–G)** IL-6R antibody was used to block IL-6 signal and the downstream signaling pathway. **(E)** Phosphorylation levels of JAK1, JAK2, TYK2, and STAT3 proteins decreased after the addition of the anti-IL-6R neutralizing antibody (*n* = 3). **(F)** The mRNA level of SOCS3 was increased, while the mRNA level of SOCS1 was decreased after blocking of CD126 (*n* = 3). **(G)** The expression of corresponding proteins in MDSCs displayed the same trend as that of mRNA after CD126 blocking. β-actin blots were used as protein loading controls (*n* = 3). *P < 0.05, **P < 0.01, ***P < 0.001.

The IL-6 signal is transduced as a result of the interaction between IL-6 and IL-6R, which includes 2 subunits, CD126 and gp130 (8). We analyzed the expression of CD126 and gp130 in primary MDSCs and found that the mRNA levels of CD126 and gp130 in primary MDSCs were higher than those in CD33^+^ and CD14^+^ controls (Figure 6A). Furthermore, the mRNA levels of CD126 and gp130 in primary MDSCs^IL-6h^ were higher than those in MDSCs^IL-6l^ (*P* = 0.010, Figure 6B). Linear regression analysis demonstrated that CD126, rather than gp130, was positively correlated with IL-6 levels (*R*^2^ = 0.6717, *P* < 0.0001, Figure 6B). Similarly, we examined expression of CD126 and gp130 in induced MDSCs, and found that these MDSCs exhibited higher mRNA levels of CD126 and gp130 than CD33^+^ controls (*P* = 0.0145, *P* = 0.0011, Figure 6C). Similar results were obtained at the protein level, in which CD126 expression was significantly enhanced, while gp130 showed no significant changes in the expression (Figure 6D).

An anti-IL-6R (CD126) neutralizing antibody was used to block the interaction between IL-6 and IL-6R in MDSCs to study the effect of elevated CD126 on the JAK/STAT pathway. Phosphorylation levels of JAK1, JAK2, TYK2, and STAT3 proteins decreased after the addition of the anti-IL-6R neutralizing antibody (Figure 6E). This result indicates that CD126 plays a significant role in IL-6-dependent activation of the JAK/STAT pathway. Furthermore, the mRNA level of SOCS3 increased, while the mRNA level of SOCS1 decreased after blocking CD126 (*P* = 0.0318, *P* = 0.0190, Figure 6F). The expression of corresponding proteins in MDSCs displayed the same trend as that of mRNA after CD126 blocking (Figure 6G). These results indicate that suppressed expression of SOCS3 is significantly correlated with CD126 upregulation, which induces long-term activation of the JAK/STAT pathway.

### Soluble CD126-Mediated IL-6 *Trans*-Signaling Regulated IL-6 Dependent SOCS3 Suppression and Sustained Activation of the JAK/STAT Pathway in MDSCs

**Figure 7:**
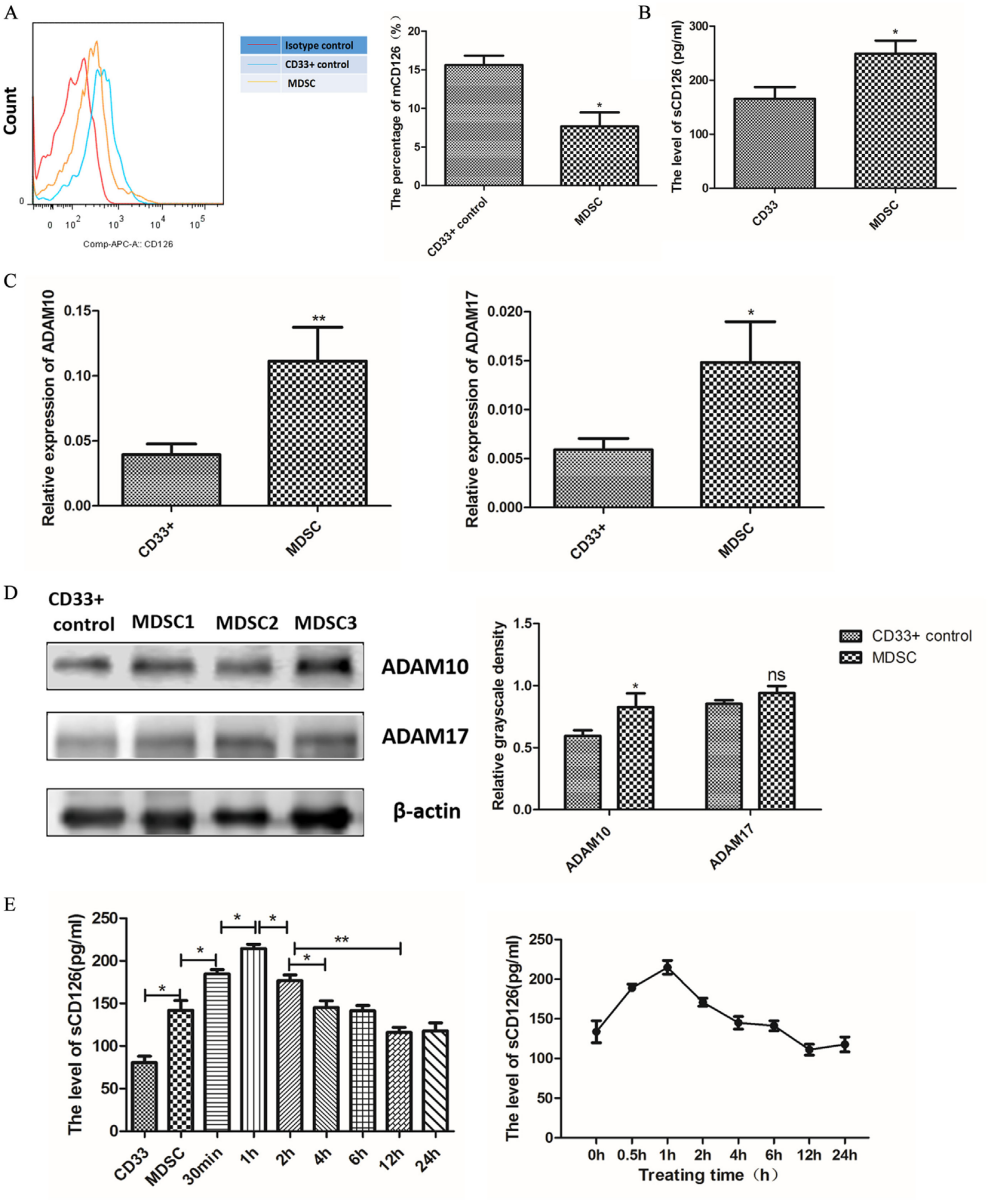

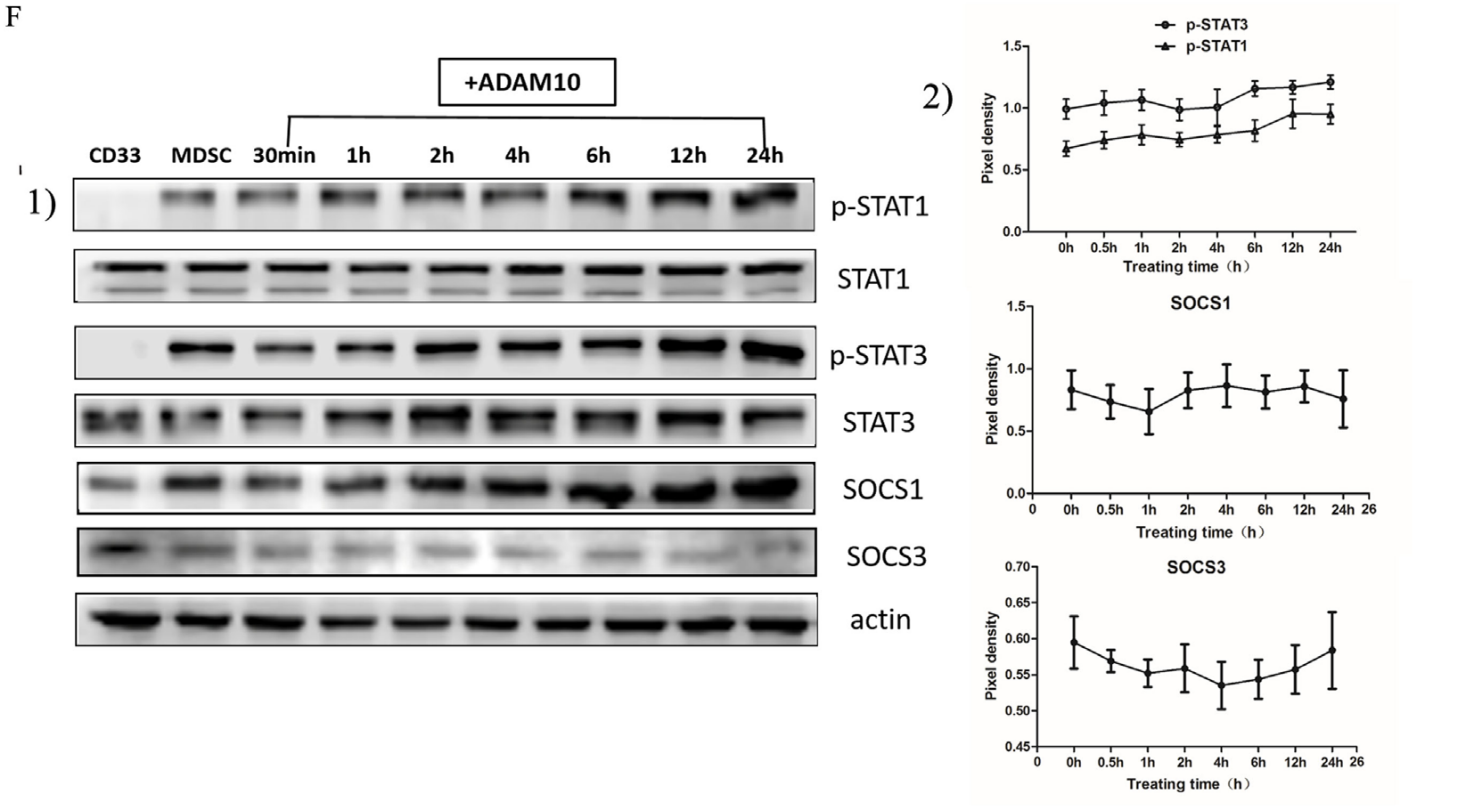
Soluble CD126-mediated interleukin-6 (IL-6) *trans*-signaling regulated IL-6 dependent suppressor of cytokine signaling 3 (SOCS3) suppression, and sustained activation of the JAK/STAT pathway in myeloid-derived suppressor cells (MDSCs). **(A)** Flow cytometry showed that MDSCs expressed lower levels of membrane-bound CD126 than those in CD33^+^ controls (*n* = 5). **(B)** The soluble CD126 secretion increased in MDSCs using Elisa assay method (*n* = 5). **(C)** RT-PCR method was used to detect ADAM10 and ADAM17 expression. The mRNA levels of ADAM10 and ADAM17 were clearly enhanced in MDSCs compared to in CD33^+^ controls (*n* = 3). **(D)** Protein expression of ADAM10, but not ADAM17, was significantly increased (*n* = 3). **(E)** MDSCs were then treated with exogenous recombinant ADAM10 protein, and the change in soluble CD126 at different time points was measured. The level of soluble CD126 in MDSCs was increased at 30 min and decreased to pre-treatment levels at 2 h (*n* = 3). [**(F)**, 1] The activation of STAT and SOCS in MDSCs at different time points after adding exogenous ADAM10 were detected. We found that the levels of p-STAT1 and p-STAT3 proteins increased after ADAM10 treatment in MDSCs. A slight increase in SOCS1 and decrease in SOCS3 protein were also detected after adding ADAM10 in MDSCs. [**(F)**, 2] Quantification of immunoblot density was performed by normalizing the density of each band to STAT1, STAT3 or β-actin (*n* = 3). *P < 0.05; **P < 0.01, ***P < 0.001.

Signaling through membrane-bound and soluble IL-6R (CD126) is known as the *cis*- and *trans*-mediated signaling pathways, respectively (29). To investigate which type of CD126 mainly regulates IL-6-dependent activation of the JAK/STAT pathway, we measured the levels of membrane-bound and soluble CD126 in MDSCs. The results showed that MDSCs expressed lower levels of membrane-bound CD126 (7.667 ± 1.808 vs. 15.63 ± 1.200%, *P* = 0.0214, Figure 7A), but generated more soluble CD126 than those in CD33^+^ controls (249.1 ± 24.35 vs. 165.6 ± 21.83 pg/mL, *P* = 0.0236, Figure 7B). These results demonstrate that soluble CD126 is significantly increased in MDSCs and may play major roles in suppressing SOCS3 expression and activating the JAK/STAT pathway in MDSCs.

To determine if soluble CD126 regulates SOCS3 expression and activation of the JAK/STAT pathway, we added ADAM proteases to the MDSC culture system *in vitro*. ADAM proteases, particularly ADAM10 and ADAM17, can induce shedding of membrane CD126 (30). We firstly detected the expression of ADAM10 and ADAM17 in MDSCs by RT-PCR and western blotting and found that the mRNA levels of ADAM10 and ADAM17 were clearly enhanced in MDSCs compared to those in CD33^+^ controls (*P* = 0.0064; *P* = 0.0297, Figure 7C). But at the protein level, exclusively ADAM10 rather than ADAM17 significantly increased (Figure 7D). We then treated MDSCs with exogenous recombinant ADAM10 protein and measured the levels of soluble CD126 at different time points. The level of soluble CD126 in MDSCs significantly increased at 30 min (184.7 ± 5.066 vs. 142.0 ± 11.50 pg/mL, *P* = 0.0273) and decreased to pre-treatment levels at 2 h (125.1 ± 9.050 pg/mL, Figure 7E).

We next detected the activation of STAT and SOCS in MDSCs at different time points after adding exogenous ADAM10. We found that the levels of phosphorylated STAT1 and STAT3 proteins increased after ADAM10 treatment in MDSCs (Figure 7F). A slight increase in SOCS1 and decrease in SOCS3 protein were also detected after adding ADAM10 in MDSCs (Figure 7F). These results revealed that ADAM10 promotes the suppression of SOCS3 expression and phosphorylation of STAT proteins in MDSCs. This indicates that IL-6 *trans*-signaling is predominately mediated by soluble CD126 to regulate IL-6-dependent SOCS3 suppression and sustained activation of the JAK/STAT pathway in MDSCs, as well as coordinates the differentiation and immunosuppressive activity of MDSCs in breast cancer.

## Discussion

Multiple immunocytes recruited into the tumor microenvironment play pivotal roles in tumorigenesis (31). However, MDSCs represent a specific subset of heterogeneous immunosuppressive cells that enable cancer cells to escape immune surveillance and inhibit the host immune system attack on cancer cells (32). Bronte et al. recommended the characterization standards and nomenclature of MDSCs and indicated that MDSCs are often divided into two subtypes in humans: PMN-MDSCs and MO-MDSCs (5). In addition to these MDSCs subtypes, the eMDSC subtype is marked with Lin^−^HLA-DR^−^CD33^+^ and comprised of more immature progenitors than M-MDSCs and PMN-MDSCs (5). However, the MDSC subset is tumor-dependent. Previous studies of breast cancer examined MDSCs in mouse models rather than in humans because of the uncertainty of cell phenotypes and complicated regulatory mechanisms in human MDSCs (33–35). Determining the precise phenotype of breast cancer MDSCs in humans improves the understanding of the crosstalk between cancer cells and the microenvironment in the initiation and progression of breast cancer.

In our previous study, we identified a subset of poorly differentiated eMDSCs in breast cancer displaying potent suppression of T cells *in vitro* and *vivo* (6). As a pan-myeloid marker, CD33 is expressed earlier and more extensively in the myeloid lineage, and we found that CD33^+^HLA-DR^−^ cells rather than CD14^+^HLA-DR^−^ cells and CD11b^+^HLA-DR^−^ cells were increased in patient blood samples compared to in healthy donor blood samples (6). We further detected the expression of a series of markers of myeloid linage, including HLA-DR, CD15, CD14, CD13, and CD11b. We confirmed low expression of HLA-DR and CD14, as well as negative expression of CD15 in breast cancer MDSCs. Additionally, both CD13 and CD11b expressed on breast cancer MDSCs, however, non-specific staining on the cancer cells, endothelial cells, and fibroblasts significantly interfered with the specific staining on MDSCs which were consistent with the previous reports (36, 37) (Figures S1B–C in Supplementary Material). Therefore, we defined the phenotype of CD45^+^CD13^+^CD33^+^CD14^−^CD15^−^ to precisely distinguish breast cancer MDSCs.

In this study, we demonstrated the positive correlations between MDSCs *in situ* and numbers of metastatic lymph nodes, tumor volume, pathological stage, and histology grade. Furthermore, we confirmed the negative correlation between MDSCs and OS in breast cancer patients and found that patients with more MDSCs showed worse clinical outcomes. Similar findings were reported in other tumors, such as in digestive system malignant tumors (38), prostate cancer (39), and advanced melanoma (40). Our results indicate that MDSCs are unfavorable prognostic factors in breast cancer patients.

Numerous cytokines have been reported to recruit MDSCs in cancer tissues, such as IL-1β, IL-6, IL-4, macrophage colony-stimulating factor, and granulocyte macrophage colony-stimulating factor (3, 14, 32). Among these tumor-derived cytokines, IL-6 has been proposed to be an efficient MDSCs inducer in solid tumors, such as esophageal cancer, prostate cancer, and melanoma (9, 28, 41–43). Circulating CD11b^+^CD14^+^HLA^−^DR^−^ cells were found to be significantly increased in esophageal cancer and were associated with circulating IL-6 levels (9). IL-6 induces MDSCs generation, and inhibition of IL-6 abrogates generation of MDSCs in tumor-bearing mice (13, 42). In this study, we evaluated the correlation between tumor-derived IL-6 and MDSC infiltration in 253 paraffin-embedded primary breast tissues and 20 fresh breast cancer tissues. We found that more MDSCs infiltrated IL-6 high-expressing cancer tissues, and that tumor-derived IL-6 displayed a strong positive correlation with the number of infiltrating MDSCs *in situ* at both the mRNA and protein levels. Furthermore, we demonstrated that tumor-derived IL-6 was essential for MDSCs amplification and function *in vitro*, including promoting T cells apoptosis, inhibiting T cell proliferation, decreasing IFN-γ secretion, and increasing IL-10 production. Therefore, determining the detailed molecular mechanisms that regulate IL-6-dependent recruitment and amplification of MDSCs in breast cancer may help screen for potential therapeutic targets to eradicate MDSCs and reverse MDSCs-mediated immune tolerance in breast cancer patients.

Interleukin-6 signals are transduced *via* the JAK/STAT signaling pathway in most cell types (44–46). Aberrant activation of the JAK/STAT signaling pathway in MDSCs has been reported in pancreatic cancer (15) and multiple myeloma (47). Physiologically, cytokine signal transduction can be switched off by SOCS proteins (48). Therefore, the activation of the JAK/STAT signaling pathway is rapid and reversible in normal cells. However, defects in SOCS expression frequently occur in malignant cells (16, 19), causing sustained phosphorylation of key proteins along the JAK/STAT signaling pathway (16, 17). In this study, we found that tumor-derived IL-6 triggers the differentiation and immunosuppressive activity of MDSCs. This was accompanied by sustained activation of the JAK/STAT signaling pathway, which led to phosphorylation of the STAT1, STAT3, JAK1, JAK2, and TYK2 proteins. Furthermore, the activation of the JAK/STAT signaling pathway in MDSCs was persistent, and lasted longer than that in normal myeloid controls. Accordingly, significant suppression of SOCS3 at both the RNA and protein levels was observed in MDSCs. Therefore, significant defects in the SOCS feedback loop may participate in the regulation of IL-6-dependent, sustained activation of the JAK/STAT signaling pathway in MDSCs.

The SOCS protein family consists of SOCS1–7 and CIS, which are divided into three subgroups: CIS and SOCS1–3, SOCS4/5, and SOCS6/7. CIS and SOCS1–3 are associated with the control of cytokine signaling, whereas the SOCS4–7 subgroup regulates the growth factor-induced receptor tyrosine kinase signaling (19). As reported previously, the expression of SOCS proteins is rapidly upregulated by IL-6, among which SOCS3 is the most important, and in turn, inhibits IL-6 cytokine signaling (48, 49). Numerous reports showed that SOCS3 defects are responsible for sustained IL-6/STAT3 signaling in human cancers (16, 50, 51). However, few studies have examined the expression of SOCS3 in immune cells. SOCS3 can also regulate the activation and differentiation of naïve CD4^+^ T cells, preferentially by promoting Th2 and inhibiting Th1 differentiation (52). In addition, SOCS3 can regulate the activation of DCs and polarization of macrophages (53, 54). Regarding MDSCs, recent studies demonstrated that SOCS3 negatively regulates the development and function of MDSCs *via* inhibition of STAT3 activation in prostate cancer (25). SOCS3-deficient mice showed elevated Gr-1^+^CD11b^+^ MDSCs in tumors and exhibited heightened STAT3 activation (25). Consistent with the above results, we found that SOCS3 was significantly decreased in primary breast cancer MDSCs and induced MDSCs and was significantly correlated with sustained activation of the JAK/STAT signaling pathway and enhanced T cells immunosuppression in MDSCs. Furthermore, in a co-culture system *in vitro*, we demonstrated that suppressed expression of SOCS3 was initiated by IL-6. This explains the phenomenon observed in our previous study, which showed that cancer-derived IL-6-induced T cell suppression in primary MDSCs by activating STAT3-dependent, nuclear factor-κB-mediated long-term IDO overexpression (7). Thus, SOCS3 defects may be the main cause of IL-6-induced persistent activation of the JAK/STAT signaling pathway and consequent enhanced differentiation and immunosuppressive activity of MDSCs.

Interestingly, in this study, we also found synchronous yet opposing changes in SOCS1 and SOCS3 expression at both the mRNA and protein levels. In contrast to SOCS3, SOCS1 expression was dramatically increased by IL-6-dependent sustained activation of the JAK/STAT signaling pathway in MDSCs. Both SOCS1 and SOCS3 have been demonstrated to inhibit phosphorylation of gp130, STATs, and JAK proteins along the JAK/STAT signaling pathway (48, 49). However, for IFN-α and IFN-γ secretion, SOCS1 is not as efficient as SOCS3 in inhibiting IL-6-dependent activation of the JAK/STAT signaling pathway (55). SOCS3 is associated with specific phosphotyrosine motifs within the activated IL-6 receptor gp130 (56–58), which directly inhibit the catalytic domains of JAK1, JAK2, and TYK2 (59). This may explain the relative specificity of SOCS3 in inhibiting IL-6 pathways. Therefore, the increase in SOCS1 may be a consequence of sustained IL-6 stimulation, which is consistent with the results of other studies (60, 61).

We further demonstrated that IL-6-induced inhibition of SOCS3 and activation of the JAK/STAT pathway was correlated with the elevated expression of CD126 *via* the IL-6 *trans*-signaling pathway. The IL-6 signaling complex assembly is composed of IL-6, CD126, and the shared signaling receptor gp130. CD126 exists in two forms, membrane-bound and soluble CD126. IL-6 signal transduction *via* membrane-bound CD126 is known as the *cis*-signaling pathway, while signal transduction *via* soluble CD126 is known as the *trans*-signaling pathway (8). The IL-6 *cis*-signaling pathway is mainly limited to hepatocytes, megakaryocytes, neutrophils, and certain T cell subsets (62). In contrast, the IL-6 *trans*-signaling pathway can potentially stimulate all types of cells that do not express membrane-bound IL-6R. During IL-6 *trans*-signaling, the soluble form of CD126 is generated either by alternative splicing or shedding of membrane-bound IL-6R, which is mediated by the metalloproteases ADAM10 and ADAM17 (29, 62, 63).

Previous studies of breast cancer indicated that MDSCs express ADAM-family proteases and IL-6Rα, which contribute to breast cancer cell invasiveness and distant metastasis through the IL-6 *trans*-signaling pathway in murine models (10). In our study, we compared the expression of IL-6R in MDSCs and found that both CD126 and gp130 were increased in MDSCs. However, while the soluble form of CD126 was increased, membrane-bound CD126 was decreased. Importantly, we reported that MDSCs express higher levels of ADAM10 as compared to that in CD33^+^ controls. These results indicate that a higher level of soluble CD126 in MDSCs may be derived from enhanced shedding of membrane-bound IL-6R by ADAM10. To verify the effect of ADAM10, we added exogenous ADAM10 to increase the level of soluble CD126. This resulted in enhanced suppression of SOCS3 and phosphorylation of STAT1 and STAT3 in MDSCs. Although a previous study demonstrated that reduced expression of membrane-bound CD126 may result in impaired IL-6 classic signaling, followed by decreased phosphorylation of STAT3 and STAT1 (64), our results indicated that soluble CD126-mediated IL-6 *trans*-signaling pathway is sufficient for IL-6 signal transduction in MDSCs. Downstream effects include persistent activation of the JAK/STAT pathway and generation of more immunosuppressive MDSCs *via* suppression of SOCS3.

Taken together, this study provides insight into the cross-talk between breast cancer cells and regulatory immunocytes in local microenvironments. In breast cancer, tumor-derived IL-6 predominantly modulates the differentiation and immunosuppressive ability of MDSCs at both the tissue and cellular levels in which the soluble CD126-mediated IL-6 *trans*-signaling pathway and SOCS3 suppression are the most crucial molecular events orchestrating IL-6-dependent sustained activation of the JAK/STAT pathway in breast cancer MDSCs. Therefore, blocking the IL-6 signaling pathway is a promising therapeutic strategy for eliminating and inhibiting MDSCs, as well as reversing MDSCs-mediated immune escape in breast cancer.

## Ethics Statement

This study was approved by the Medical Ethics Committee of Tianjin Medical University. All experiments were performed in accordance with the principles of the Declaration of Helsinki. Written consents were obtained from all patients and healthy donors.

## Author Contributions

MJ performed the research, data analyses, and wrote the manuscript. JC and RZ performed parts of the research and commented on manuscript. WZ, YY, PL, and WY performed parts of the research and data analyses. FW and XR contributed clinical information and samples for the study. JY designed the study and commented on manuscript.

## Conflict of Interest Statement

The authors declare that the research was conducted in the absence of any commercial or financial relationships that could be construed as a potential conflict of interest.
