# Supplementary material for: Interleukin-6 Trans-Signaling Pathway Promotes Immunosuppressive Myeloid-Derived Suppressor Cells via Suppression of Suppressor of Cytokine Signaling 3 in Breast Cancer
Source: Front Immunol. 2017 Dec 15;8:1840. doi: 10.3389/fimmu.2017.01840 (PMC5736866; doi:10.3389/fimmu.2017.01840)
Supplement: Supplementary file 1 [file Image_1.PDF]

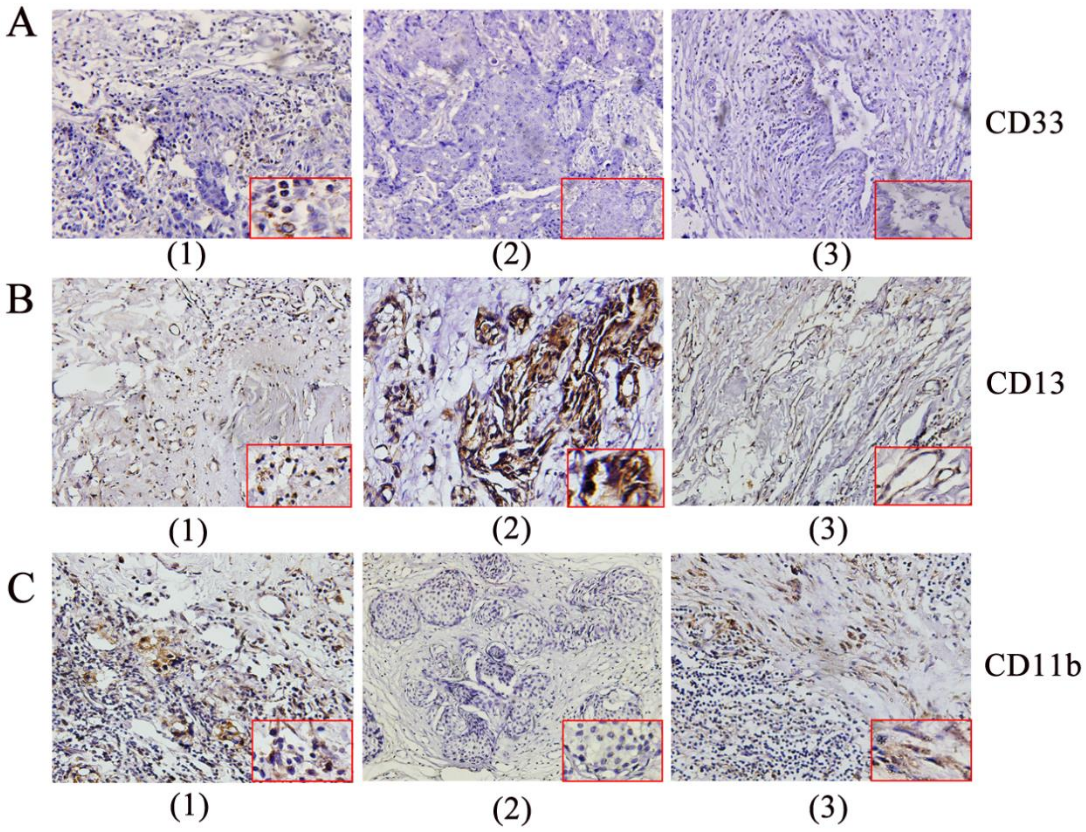

Supplemental Figure 1. 50 breast cancer patients were selected for IHC staining of CD33, CD13 and CD11b. Original magnification×200. $CD13^{+}$ and $CD11b^{+}$ cells were scattered in the stroma of breast cancer tissues similar to the distribution of $CD33^{+}$ cells (Fig1A-1,1B-1,1C-1). But CD33 did not express in breast cancer cells (Fig. 1A-2) and endothelial cells (Fig. 1A-3). However, CD13 expressed in breast cancer cells (Fig. 1B-2) and endothelial cells (Fig. 1B-3). CD11b did not express in breast cancer cells (Fig. 1C-2) but in fibroblasts (Fig. 1C-3). Thus, CD13 and CD11b may not be regarded as specific markers of breast cancer MDSCs.
